# Fibre elongation requires normal redox homeostasis modulated by cytosolic ascorbate peroxidase in cotton (*Gossypium hirsutum*)

**DOI:** 10.1093/jxb/erw146

**Published:** 2016-04-17

**Authors:** Kai Guo, Xueqiong Du, Lili Tu, Wenxin Tang, Pengcheng Wang, Maojun Wang, Zhen Liu, Xianlong Zhang

**Affiliations:** National Key Laboratory of Crop Genetic Improvement, Huazhong Agricultural University, Wuhan 430070, Hubei, China

**Keywords:** Cytosolic ascorbate peroxidase, fibre elongation, *Gossypium hirsutum*, hydrogen peroxide, oxidative stress, redox homeostasis.

## Abstract

High-quality cotton fibre equates to a more comfortable textile. Fibre length is an important index of fibre quality. Hydrogen peroxide (H_2_O_2_) acts as a signalling molecule in the regulation of fibre elongation. Results from *in vitro* ovule culture suggest that the alteration of fibre cell H_2_O_2_ levels affects fibre development. Ascorbate peroxidase (APX) is an important reactive oxygen species (ROS) scavenging enzyme, and we found that *GhAPX1A_T_/D_T_* encoded one member of the previously unrealized group of cytosolic APXs (cAPXs) that were preferentially expressed during the fibre elongation stage. Transgenic cottons with up- and down-regulation of *GhAPX1A_T_/D_T_* were generated to control fibre endogenous levels of H_2_O_2_. Suppression of all *cAPX* (IAO) resulted in a 3.5-fold increase in H_2_O_2_ level in fibres and oxidative stress, which significantly suppressed fibre elongation. The fibre length of transgenic lines with over-expression or specific down-regulation of *GhAPX1A_T_/D_T_* did not show any obvious change. However, the fibres in the over-expression lines exhibited higher tolerance to oxidative stress. Differentially expressed genes (DEGs) in fibres at 10 days post-anthesis (DPA) of IAO lines identified by RNA-seq were related to redox homeostasis, signalling pathways, stress responses and cell wall synthesis, and the DEGs that were up-regulated in IAO lines were also up-regulated in the 10 DPA and 20 DPA fibres of wild cotton compared with domesticated cotton. These results suggest that optimal H_2_O_2_ levels and redox state regulated by cytosolic APX are key mechanisms regulating fibre elongation, and dysregulation of the increase in H_2_O_2_ induces oxidative stress and results in shorter fibres by initiating secondary cell wall-related gene expression.

## Highlight

Optimal H_2_O_2_ levels and redox state regulated by cytosolic ascorbate peroxidase are key mechanisms regulating fibre elongation in cotton.

## Introduction

Cotton fibre is an important textile material and plays an important role in the world economy. The fibre is a single cell that is initiated from the outermost cell layer of the cotton ovule and undergoes five overlapping developmental stages, including differentiation, elongation, transition, secondary cell wall thickening and maturity, and then is converted to valuable lint (Lee *et al.*, 2007; Qin and Zhu, 2011). The mature fibre length is one of the most important indexes for the evaluation of fibre quality, and the final fibre length is mainly determined by the duration of the elongation stage. The secondary cell wall synthesis stage mainly determines other fibre quality indexes such as the micronaire, strength and elongation ratio, among others (Lee *et al.*, 2007; Stiff and Haigler, 2013). Thus, the time to establish the transition stage, from the elongation stage to the secondary cell wall synthesis stage, could affect the fibre development and quality.

Three important factors determine fibre cell elongation, namely cell osmotic pressure, cell wall loosening and synthesis of the structural molecules necessary for cell elongation. Sugar, organic acids and potassium maintain the osmotic pressure of the cell and drive horizontal and longitudinal elongation (Ruan *et al.*, 2001). Secondary cell wall thickening occurs through the deposition of cellulose, and once the synthesis of the secondary cell wall is initiated, cell elongation will stop. High levels of H_2_O_2_ are accumulated in the transition stage from elongation to secondary cell wall synthesis, inducing an oxidative stress that promotes secondary cell wall initiation and cellulose synthesis (Potikha *et al.*, 1999; Kurek *et al.*, 2002). Therefore, H_2_O_2_ may act as an important factor in regulating fibre development by controlling its levels during different developmental stages.

ROS are continuously produced in plants as by-products of aerobic metabolism, whereby oxygen is reduced to water. H_2_O_2_ is the major active type of ROS because of its relative stability and permeability (Apel and Hirt, 2004). Recent biochemical and genetic studies have confirmed that H_2_O_2_ is a signalling molecule that mediates many biology processes such as stomatal closure, root hair initiation and elongation, pollen tube growth, and lignification, among others (Pei *et al.*, 2000; Foreman *et al.*, 2003; Rentel *et al.*, 2004; Suzuki *et al.*, 2011). NADPH oxidase-dependent generation of ROS is important for the regulation of cell elongation, including cell polarized growth and diffuse growth (Suzuki *et al.*, 2011). For polarized growth, such as in root hairs and pollen tubes, there are steep gradients of ROS accompanied by an enrichment of Ca^2+^ in the cell tip. During diffuse growth, such as during the growth of the hypocotyl and trichome cells, ROS appear to modify cell wall polysaccharides and loosen interactions to promote cell extension (Marino *et al.*, 2012).

Fibre cell elongation represents a linear cell growth mode with characteristics of both tip growth and diffuse growth (Qin and Zhu, 2011). The regulatory role of ROS has been explored in cotton fibre elongation. Treatment of ovule cultures with 1 µM diphenyliodonium (DPI) inhibited ROS generation and completely abrogated fibre elongation. The application of optimal concentrations of H_2_O_2_ promoted fibre cell extension in the ovule culture system via the ETH (ethylene) signalling pathway (Li *et al.*, 2007; Qin *et al.*, 2008). High concentrations of H_2_O_2_ could induce the nuclear localization of GhWLIM1a, which functioned as a transcription factor to activate the expression of genes involved in phenylpropanoid biosynthesis and lignin biosynthesis to construct the secondary cell wall (Han *et al.*, 2013). Previously, we also found that H_2_O_2_ was regulated by *GhCaM7* to modulate fibre elongation, and increased concentrations of H_2_O_2_ promoted early fibre elongation via the overexpression of *GhCaM7* (Tang *et al.*, 2014). Based on these results, H_2_O_2_ contributes to fibre elongation and secondary cell wall synthesis in a manner that is dependent on the H_2_O_2_ content during the fibre development stage. Therefore, we speculated that the H_2_O_2_ content during the transition stage, from the elongation to the secondary cell wall synthesis stage, might regulate the fibre development pattern.

To maintain plant cell viability, antioxidant and ROS-scavenging enzymes, including ascorbate peroxidase (APX), catalase (CAT), superoxide dismutase (SOD) and glutathione peroxidase (GPX), are highly dynamic and redundant. APX was first discovered in chloroplasts in 1979 (Groden and Beck, 1979; Kelly and Latzko, 1979), and is a plant-specific haem-containing peroxidase located in the cytosol, chloroplast, mitochondrion and peroxisome (Shigeoka *et al.*, 2002; Foyer and Noctor, 2011). APX maintains the balance of the cellular redox state for normal plant growth by utilizing reduced ascorbic acid (ASA) as its specific electron donor to reduce H_2_O_2_ to H_2_O, with the concomitant generation of dehydroascorbic acid (DHA) (Shigeoka *et al.*, 2002). GhAPX1 is one of the proteins that was discovered in the elongating fibre; it accumulates to high levels during the fibre elongation stage of *G. hirsutum* Xuzhou142, and it can be induced by H_2_O_2_ and ETH (Li *et al.*, 2007).

Many environmental factors can significantly damage the fibre length and quality, such as extreme weather, drought and nutrient deficiency (Hodges and Constable, 2010; Padmalatha *et al.*, 2012). Such environmental stresses can induce high levels of H_2_O_2_ accumulation (Foyer and Noctor, 2011). Therefore, it is important to understand the mechanism underlying fibre elongation and the role of H_2_O_2_ in fibre development and secondary cell wall formation. In this study, we explored the relationship between H_2_O_2_ and fibre elongation. Using gain- and loss-of-function analysis by up- and down-regulation of *GhAPX1A*_*T*_ */D*_*T*_ to control H_2_O_2_ levels in the fibre cells, we found that RNA interference of cytosolic *APX* genes induced oxidative stress during the fibre elongation stage and suppressed fibre elongation while also initiating secondary cell wall-related gene expression during the elongation stage. These results suggest that fibre elongation requires optimal H_2_O_2_ levels achieved via a fine spatiotemporal modulation of redox homeostasis.

## Materials and methods

### Plant material

Cotton plants *Gossypium hirsutum* acc. YZ1, TM-1, Xuzhou142, *Xuzhou142 fuzzless-lintless* and T586 were grown in an experimental field at Huazhong Agricultural University in Wuhan, Hubei province, PR China. Bolls were marked on the day of anthesis (0 days post-anthesis (DPA)). Ovules and fibres at different developmental stages were stripped from bolls, immediately immersed in liquid nitrogen, and then stored at –70 °C until use. Fibres were lightly knocked off the ovules with a pestle in liquid nitrogen, and then ground into powder. Roots, leaves and hypocotyls were collected from seedlings with two true leaves in the greenhouse.

### Ovule culture

Bolls were collected at 0 DPA or 1 DPA, sterilized in 0.1% HgCl_2_ for 20min and washed with sterilized double-distilled water five times. Ovules from bolls were floated on the surface of ovule liquid culture medium with or without different chemical applications in 50ml flasks, and then incubated at 30 °C in the dark (Tan *et al.*, 2013). Filter-sterilized H_2_O_2_ was dissolved in sterile double-distilled water. The NADPH oxidase inhibitor diphenyliodonium chloride (DPI; cat. no. D2926, Sigma-Aldrich, St Louis, MO, USA) was dissolved in sterile 0.1% dimethyl sulfoxide (DMSO). After 8–10 days of culture, the fibre length was determined. Cultured ovules were boiled in double-distilled water until the fibre straightened. The length of the fibre was measured manually with a ruler after the fibres were arranged gently on the filter paper into straight lines (Tang *et al.*, 2014). At least ten ovules were measured for each replicate. At least three replicates were assessed for the different treatments, and the data were analysed using Student’s *t* test or Duncan’s multiple comparisons.

### Gene cloning and sequence analysis

The 5′-UTR and 3′-UTR fragments were cloned based on the sequence of EF432582.1 using the cDNA of *G. hirsutum* YZ1 fibre as the template and the SMART RACE cDNA amplification kit (Clontech, Mountain View, CA, USA). The nucleotide sequences and amino acid sequences of APX were downloaded from NCBI (http://www.ncbi.nlm.nih.gov/guide/), TAIR (http://www.arabidopsis.org/) and phytozome 9.1 (http://www.phytozome.net/cotton.php). Sequence alignment was performed with Clustalx 1.8, and phylogenetic trees were constructed with MEGA 5.1.

### Vector construction, plant transformation and Southern blotting

To generate an overexpression construct, the cDNA of *GhAPX1A*_*T*_ was amplified and inserted into the 35S overexpression vector pK2GW7.0 (Ghent University) using Gateway technology (http://www.plantgenetics.rug.ac.be/gateway/). To make the RNAi constructs, the 3′-UTR sequence and the ORF of *GhAPX1A*_*T*_ were amplified by PCR (for primers with added attB1 and attB2 adaptors, see Supplementary Table S1 at *JXB* online), and the PCR products were inserted into the plasmid pHellsgate4 using the BP reaction to generate the *GhAPX1A*_*T*_ */D*_*T*_ -3′-UTR RNAi (IAU) and *GhAPX1A*_*T*_ */D*_*T*_ -ORF RNAi (IAO) constructs (Helliwell *et al.*, 2002).

All constructs were transformed into *G. hirsutum* YZ1 mediated by *Agrobacterium tumefaciens* (EHA105, LBA4404) according to previous reports (Jin *et al.*, 2006a; Jin *et al.*, 2006b). Genomic DNA was extracted and digested with *Hin*dIII, and Southern blotting was performed to determine the copy number according to Li *et al.* (2010). *NPT* II was used as the probe. λ DNA digested with *Hin*dIII was used as the marker DNA.

### Reverse transcription-PCR, quantitative real-time PCR and northern blotting

All samples were collected in liquid nitrogen and ground into powder. Total RNA was extracted according to a previously described method (Zhu *et al.*, 2005), and cDNA was synthesized with SuperScript III reverse transcriptase (Invitrogen, Carlsbad, CA, USA). RT-PCR was performed using an ABI 9700 (Applied Biosystems, Gene.Amp® PCR system 9700), and RT-qPCR was performed as previously described (Hao *et al.*, 2012) using an Applied Biosystems 7500 Real-Time PCR System. *GhUB7* (DQ116411) served as the internal control to normalize expression levels. For northern blotting, 20 µg RNA was transferred to nylon membranes (Millipore, Billerica, MA, USA). The ORF of *GhAPX1A*_*T*_ or *18S RNA* was used as the template to synthesize probes labelled with [α-^32^P]dCTP at room temperature for 4h using the Prime-a-Gene® Labeling System kit (Promega, Madison, WI, USA). Hybridization, washing and signalling detection were performed as described previously (Tu *et al.*, 2007). *Gh18S RNA* was used as the reference gene. The primers are listed in Supplementary Table S1 at *JXB* online.

### Western blotting, enzyme activity analysis

Total protein extraction was performed essentially according to a previously reported method (Deng *et al.*, 2012). Ovules or fibres (0.1g) were ground into powder with liquid nitrogen, and total protein was extracted in buffer (0.1M PBS pH 7.0, 5mM ASA, 1mM DTT, 1mM Na_2_EDTA, 10% (v/v) glycerol, 2% (m/v) PVPP-40, 0.1% (v/v) β-mercaptoethanol) on ice for 10min, followed by centrifugation at 15 600 *g* for 20min at 4 °C. The supernatant was used to determine the protein concentration and for western blotting and enzyme activity measurements.

Polyclonal antiserum against a synthetic peptide corresponding to residues 8–24 (VSEEYQKSVEKAKRKLR) of GhAPX1A_T_ (NewEast Bioscience, Wuhan, China) was collected from rabbits. Western blotting was performed as previously described (Hu *et al.*, 2011).

Four types of enzymes activities were detected at 25 °C as described previously with minor modifications (Bonifacio *et al.*, 2011). APX activity was measured by evaluating ascorbate oxidation by the decrease in absorbance at 290nm. After adding 10 µl 10mM ASA and 10 µl extraction supernatant to 970 µl reaction buffer (50mM PBS pH 7.0, 0.1mM EDTA), the reaction was started by adding 10 µl 10mM H_2_O_2_. The absorbance was recorded every 10s for a duration of 100s. Peroxidase (POD) enzyme activity was determined after adding 10 µl extraction supernatant to 980 µl reaction buffer (50mM PBS pH 7.0, 0.1mM EDTA, 50mM guaiacol), and the reaction was started by adding 10 µl 0.5M H_2_O_2_. The absorbance was recorded every 10s for a duration of 100s at 470nm. To measure catalase (CAT) enzyme activity, 25 µl extraction supernatant was added to 975 µl reaction buffer (50mM PBS pH 7.0, 0.1mM EDTA, and 12.5mM H_2_O_2_), and the reaction was started. The absorbance was recorded every 30s at 240nm for 300s. Superoxide dismutase (SOD) activity was determined via the inhibition of blueformazane production using nitroblue tetrazolium (NBT) photoreduction. Next, 345 µl reaction buffer (50mM PBS (pH 7.8, 0.1mM EDTA), 50 µl 130mM Met, 50 µl 20 µM riboflavin, and 5 µl protein extraction were mixed in the dark. Three control reaction tubes were identical to the experimental extract tubes but lacked the protein extract. The reaction was performed under bright light for 20min. The absorbance was recorded at 560nm after reaction. The SOD activity unit (U) was defined as the amount of enzyme required to inhibit 50% of the NBT photoreduction.

### Fibre quality analysis of transgenic plants

Transgenic cotton and controls plants (wild type and null control) were grown together in the experiment fields at Huazhong Agricultural University, Wuhan, Hubei province, with one plot for T3 transgenic cotton (2013) and two plots for T4 transgenic cotton (2014). Mature bolls were collected simultaneously from the same position of the plants (middle part). For one plot, three samples from the T3 generation (2013) and five samples from the T4 generation (2014) (each sample >8g) were collected from each transgenic cotton line to evaluate the fibre quality using a High Volume Instrument (HVI) (HFT9000, Premier, India) method. The data for one plot was used for the analysis by Duncan’s multiple comparisons. For immature fibre (5, 10, 15 and 20 DPA) length measurements, all bolls were collected simultaneously from the same positions of different plants. The length of the fibre was measured according to a previously reported method (Tang *et al.*, 2014). Each sample from one stage comprised three replicates. Data were analysed using Duncan’s multiple comparisons.

### Detection of H_2_O_2_ and ASA content

ROS detection in the fibre was performed using the fluorescent indicator dye 2′,7′-dichlorodihydrofluorescein diacetate (2′,7′-DCFDA; D6883, Sigma-Aldrich, USA) according to a previously reported method (Tang *et al.*, 2014). H_2_O_2_ levels were quantified according to a previously reported method with some modifications (Li *et al.*, 2007). Tissues (0.1g) were ground in 0.5ml 80% acetone, shaken for 10min at 4 °C on a rotary mixer, and then centrifuged for 10min at 4 °C and 11 000 *g*. The supernatant was assayed immediately. The supernatant (100 µl) was added to 100 µl freshly prepared 20% TiCl_4_ (v:v in 11M HCl) and mixed. Next, 200 µl NH_4_OH was added to the sample to form a hydroperoxide–titanium complex. After the reaction was completed, the complex pellet was obtained by centrifugation at 11 000 *g* for 5min. The pellet was collected and suspended in 1ml 1M H_2_SO_4_, and the absorbance was measured at 405nm. An H_2_O_2_ concentration gradient (0.1, 0.2, 0.5, 1 and 2 µM) was constructed to generate a standard curve.

ASA was detected according to a previously reported method (Kampfenkel *et al.*, 1995). Sample (0.1g) was ground to powder in liquid nitrogen and 0.5ml of 5% (w/v) sulfosalicylic acid was added. The mixture was continually homogenized on ice for 15min and was centrifuged at 13 000 *g* for 5min at 4 °C. The supernatant was immediately assayed. The reaction was conducted with a mixture of 100 µl supernatant, 24 µl 1.84M triethanolamine, 250 µl PBS (pH7.4) containing 2.5mM EDTA, and 50 µl 10mM DTT. After incubation in a water bath for 15min at 25 °C, 50 µl of 0.5% (w/v) *N*-ethylmaleimide was added to remove excess DTT. Incubation lasted for 10min at room temperature. The colour was developed after addition of 200 µl 10% (w/v) TCA, 200 µl 44% (v/v) *o*-phosphoric acid, 200 µl 4% (w/v) 2,2′-bipyridyl (CAS: 366-18-7, 14454, Sigma-Aldrich, USA; dissolved in 70% ethanol), and 100 µl 3% (w/v) FeC1_3_. The sample was mixed and incubated in a water bath for 1h at 42 °C. Subsequently, the absorbance in 560nm was measured with an EnSpire® Multimode Plate Reader (PerkinElmer, USA). Then total ASA (ASA+DHA) was detected. The reduced ASA content detection was performed as for the total ASA detection except for the same volume replacement of DTT and *N*-ethylmaleimide with distilled water. The DHA content was obtained from the content of total ASA subtracting the content of reduced ASA. Commercial ASA (A4544, Sigma-Aldrich, USA) dissolved in double distilled water was used for the calibration curve.

### RNA-seq for differentially expressed gene screening and verification

At least five bolls at 10 DPA were collected for each sample, and the samples from six lines were collected on the same day. For each line, the fibres were mixed together and ground to a powder with liquid nitrogen for RNA extraction. Total RNA was sequenced by RNA-Seq quantification analysis using an Illumina HiSeq^TM^2000 (BGI Company, Wuhan, China) (BioProject ID: PRJNA293202). *G. raimondii* transcripts and genome sequences (BioProject ID: PRJNA171262) were used as references for read mapping and gene annotation. For each sample, 12 million total reads were obtained, with 82% and 80% of the total reads mapped to the *G. raimondii* gene and genome, respectively (see Supplementary Table S2 at *JXB* online). The six samples were divided into three groups. The first group included two controls (Null and WT), the second group had two IAU lines (IAU20 and IAU22), and the third group included two IAO lines (IAO24 and IAO167). Screening of differentially expressed genes between two groups and pairwise comparisons between two groups were conducted using the NOIseq method (Tarazona *et al.*, 2011). The filtering parameters were a fold-change ≥2 and a probability ≥0.8. PCR verification of differentially expressed genes (DEGs) was performed following RNA-Seq, RNA extraction was performed again and reverse transcription was conducted to synthesize cDNA. Primers for RT-qPCR were designed based on the *G. raimondii* transcripts. For the RPKM values of cotton genes, raw data for TM-1 were downloaded from NCBI (PRJNA248163), and data for wild cottons (yucTX2090, yucTX2094, yucTX2095 and palmeriTX665) and domesticated cottons (TM-1, Maxxa, CRB252, Coker315 and CascotL7) were also downloaded from NCBI (SRA061240). The gene expression level was calculated using the RPKM method (reads per kilobase per million reads) with the following formula: RPKM (A)=10^6^×*C*/(*NL*/10^3^). RPKM (A) is the expression level of gene A, *C* is the number of reads that aligned uniquely to gene A, *N* is the total number of reads that aligned uniquely to all genes, and *L* is the number of bases of gene A.

## Results

### Alteration of H_2_O_2_ levels affected fibre elongation in ovule culture

**Fig. 1.**
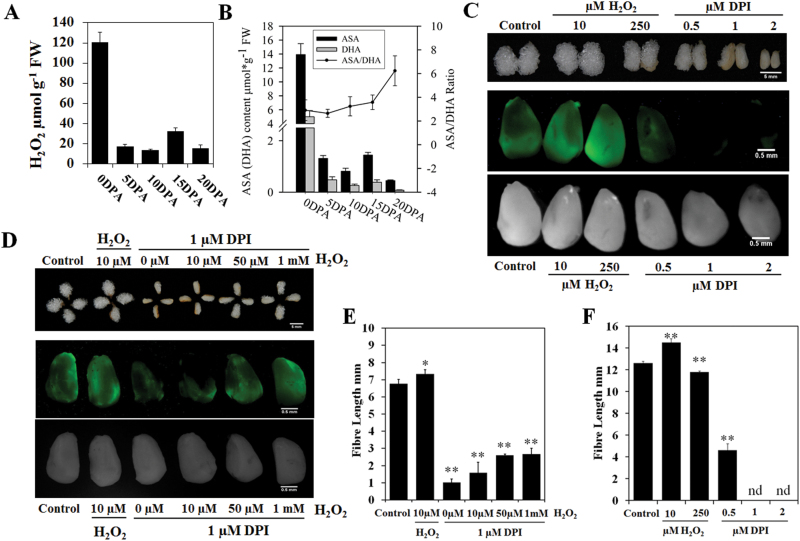
The effect of H_2_O_2_ (hydrogen peroxide) levels on fibre elongation. (A) H_2_O_2_ contents in *G. hirsutum* ovules and fibres at different development stages (mean±SE, *n*=3). 0 DPA, 0 days post-anthesis (DPA) ovules; 5–20 DPA, 5–20 DPA fibres. (B) ASA (ascorbic acid) and DHA (dehydroascorbic acid) contents and ASA/DHA ratios in 0 DPA ovules, 5 DPA, 10 DPA, 15 DPA and 20 DPA fibres (mean±SD, *n*=3). (C) *In vivo* histochemical staining for ROS in 0 DPA ovules with 10 µM 2′,7′-DCFDA after treatments for 4h with different concentrations of H_2_O_2_ and DPI. The results were observed using stereo fluorescence microscopy. The upper panel shows the fibre growth after 10 days in culture with the different treatments; the middle panel shows fluorescence images; the lower panel shows bright field images. (D) The ability of H_2_O_2_ to rescue the 1 µM DPI-induced suppression of fibre elongation. The upper panel shows fibre growth after 7 days in culture with the different treatments; the middle panel shows fluorescence images with *in vivo* histochemical staining of ROS in 0 DPA ovules with 10 µM 2′,7′-DCFDA after treatments for 4h with different concentrations of DPI and H_2_O_2_; the lower panel shows bright field images. (E, F) The fibre lengths in response to the different treatments in D and C (*n*=3; Student’s *t* test; significant differences compared with the control: *P<0.05, **P<0.01).

To reveal the relationship between levels of H_2_O_2_ and fibre elongation, we determined the spatiotemporal contents of H_2_O_2_ in the ovules or fibres from 0 DPA to 20 DPA to check whether the redox state changed during fibre development. The results showed that the H_2_O_2_ content was very high in 0 DPA ovules with fibre initials, indicating that H_2_O_2_ should be important for ovule development and fibre initiation. A small peak in H_2_O_2_ content was detected in 15 DPA fibres, which represented the transition point from the fibre elongation to secondary cell wall thickening stage (Fig. 1A). We also analysed the ASA content of developing fibres and found that ASA showed the same trend as H_2_O_2_ levels. The ASA/DHA ratios also changed during fibre development and continued to increase until the secondary cell wall thickening stage (Fig. 1B).

Then we applied the ovule culture system to test whether the redox state change could affect fibre elongation. DPI (an inhibitor of NADPH oxidase) and H_2_O_2_ with different concentrations were added to the culture medium to modify the redox state of the cell. Following the application of DPI (0.5–2 µM) to the medium, the ROS levels were significantly reduced *in vivo*, and fibre elongation was inhibited even at low concentrations (0.5 µM) (Fig. 1C, F). This observation suggested that ROS were necessary for fibre development. The fibre length was found to increase in the presence of 10 µM H_2_O_2_. The application of 250 µM H_2_O_2_ in medium significantly inhibited fibre elongation (Fig. 1C, F). *In vivo* ROS levels were also determined using a fluorescent qualitative assay utilizing 2′,7′-dichlorodihydrofluorescein diacetate (2′,7′-DCFDA). The endogenous H_2_O_2_ levels increased when H_2_O_2_ was applied to the medium, with a dramatic increase observed following the application of 250 µM H_2_O_2_ (Fig. 1C). Further experiments confirmed that H_2_O_2_ could partially rescue the fibre development inhibited by 1 µM DPI. The rescue of fibre length was more apparent as the H_2_O_2_ content increased (Fig. 1D, E). Therefore, small increases in H_2_O_2_ levels had a positive effect on promoting fibre elongation, whereas excessive H_2_O_2_ was toxic and suppressed fibre elongation. These findings also confirmed that changes in redox status could affect fibre development.

### Cytosolic *GhAPX1A*_*T*_ */D*_*T*_ was preferentially expressed during fibre elongation to control cell H_2_O_2_ levels

**Fig. 2.**
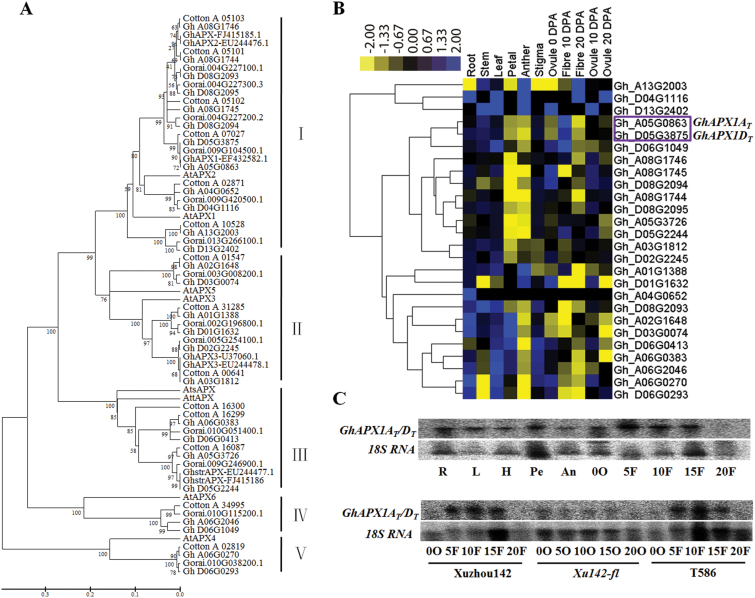
Phylogenetic and expression profile analysis of cotton *APX* (ascorbate peroxidase) genes. (A) Phylogenetic analysis of APX proteins from *G. raimondii* (*Gorai*), *G. arboreum* (*Cotton_A*), *G. hirsutum* (*Gh*), and *Arabidopsis thaliana* (*At*). The phylogenetic tree was constructed using the neighbour-joining method with pairwise deletion and bootstrap analysis with 2 000 replications. Clade I, cytosolic APX; Clade Ⅱ, peroxisomal APX; Clade Ⅲ, chloroplast APX; Clade Ⅳ, APX-R; Clade Ⅴ, APX-L. (B) Heat maps of *GhAPX* family members were constructed based on the RPKM values of RNA-seq of different tissues of *G. hirsutum* TM-1 downloaded from public databases. The purple box indicates the two sub-genomic copies of *GhAPX1.* (C) Northern blotting detection of total expression levels of *GhAPX1A*_*T*_ */D*_*T*_ in different tissues of *G. hirsutum*. R, root; L, leaf; H, hypocotyl; Pe, petal; An, anther; 0O–20O, 0 DPA–20 DPA ovule; 5F–20F, 5 DPA–20 DPA fibre.

APX was the major H_2_O_2_ scavenging enzyme. Treatments with H_2_O_2_ or ETH increased total APX activity proportionally, followed by extended fibre cell elongation (Li *et al.*, 2007; Yang *et al.*, 2008). Therefore, we decided to alter the H_2_O_2_ levels in elongating fibres by modulating the APX expression levels. First, we analysed the APX family in three types of cotton because the genomic sequences of the diploid cotton *G. raimondii* (D5), the diploid cotton *G. arboretum* (A2) and the allotetraploid cotton *G. hirsutum* TM-1 ((AD)1) are available (Paterson *et al.*, 2012; Li *et al.*, 2014; Zhang *et al.*, 2015). The phylogenetic analysis with amino acid sequences of APXs of cotton and *Arabidopsis thaliana* revealed that the family was classified into five clades (Fig. 2A). Cytosolic APX was the largest APX family subgroup. *GhAPX1* belonged to Clade I and encoded a cytosolic APX (Fig. 2A). To identify which members were preferentially expressed in the fibre, RNA-seq raw data (BioProject ID: PRJNA248163) for different tissues of *G. hirsutum* TM-1 were downloaded, and the reads were mapped to the TM-1 genome to calculate the RPKM values of the genes. A heat map was constructed based on the RPKM values of the *APX* members. The expression cluster revealed that two homologues of *GhAPX1*, *Gh_A05G0863* (A_T_ subgenome) and *Gh_D05G3875* (D_T_ subgenome), were preferentially expressed in fibres and exhibited expression peaks in 10 DPA fibres (Fig. 2B). According to the RPKM values of the *APX* expression profiles, *Gh_A05G0863* (RPKM 833) and *Gh_D05G3875* (RPKM 432) had higher expression levels compared with the others (see Supplementary Table S3 at *JXB* online). Northern blotting results confirmed that total *GhAPX1A*_*T*_ */D*_*T*_ were preferentially expressed during the fast fibre elongation stage but were not expressed in *Xuzhou142 fuzzless-lintless* (*Xu142-fl*) mutant ovules (Fig. 2C), consistent with a previous report (Li *et al.*, 2007).

Excluding *GhAPX1A*_*T*_ */D*_*T*_, some other cytosolic *APX*s exhibited moderate expression levels in 10 DPA fibres. The RPKM values of homologues of *Gorai.004G227100.1* (*G. raimondii*) in 10 DPA fibres of *G. hirsutum* were 111 (*Gh_A08G1746*) and 27 (*Gh_D08G2095*), respectively. The RPKM values of homologues of *Gorai.004G227300.3* (*G. raimondii*) in 10 DPA fibre were 40 (*Gh_A08G1744*) and 10 (*Gh_D08G2093*), respectively (see Supplementary Table S3).

### Expression levels and enzyme activities of APX were altered in transgenic lines

Because *GhAPX1A*_*T*_ */D*_*T*_ were the members with the highest expression levels during the fast elongation stage of fibres compared with the other APXs, we modulated *GhAPX1A*_*T*_ */D*_*T*_ expression levels by transforming cotton with an overexpression construct driven by the CaMV35S promoter (OA) and two RNAi constructs. For the RNAi constructs, a 155-bp 3′-UTR fragment was selected for specific interference of *GhAPX1A*_*T*_ */D*_*T*_ (IAU), and a 753-bp ORF sequence targeting all *cAPX*s was selected for suppression of cytosolic *APX*s (IAO). Twenty-four independent transgenic overexpression lines (OA), 22 independent specific RNAi lines (IAU) and 13 independent conserved sequence (ORF) RNAi lines (IAO) were developed (T0 generation; Supplementary Table S4 at *JXB* online). After screening by Southern blotting and expression level detection of the T0, T1, T2 and T3 generations, we selected nine lines (four OA lines, three IAU lines and two IAO lines) with low insertion copy numbers for further analysis (see Supplementary Fig. S1 and Supplementary Table S4 at *JXB* online).

**Fig. 3.**
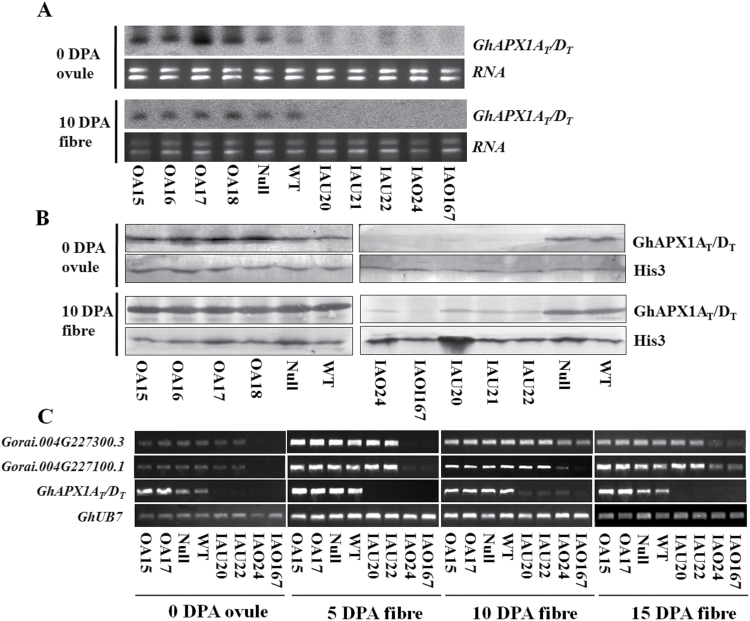
The expression levels and protein contents of APX were analysed in transgenic ovules and fibres. (A) Detection of *GhAPX1A*_*T*_ */D*_*T*_ expression level by northern blotting. The ORF sequence of *GhAPX1A*_*T*_ was used as the probe. (B) Detection of GhAPX1A_T_/D_T_ proteins by western blotting. Anti-histone3 (His3) (Abcam, San Francisco, CA, USA) was used as the reference. (C) RT-PCR analysis of cytosolic *APX* transcript levels during different developmental stages. OA15/16/17/18, individual over-expression lines; IAU20/21/22, individual *GhAPX1A*_*T*_ */D*_*T*_ RNAi lines; IAO24/167, individual cytosolic *APXs* RNAi lines; Null, transgenic negative control; WT, wild type cotton YZ1.

Transcript levels were determined by northern blotting. For the OA lines, the expression levels of *GhAPX1A*_*T*_ */D*_*T*_ were up-regulated in 0 DPA ovules but were not increased compared with WT levels in 10 DPA fibres. Native *GhAPX1A*_*T*_ */D*_*T*_ was expressed at high levels during the elongation stage, and therefore overexpression had no effect on expression levels. For both types of RNAi lines, *GhAPX1A*_*T*_ */D*_*T*_ levels were suppressed in both 0 DPA ovules and 10 DPA fibres (Fig. 3A). Protein levels detected by western blotting in transgenic lines were consistent with the transcript levels (Fig. 3B). To assess whether the expression levels of other *APX* members were modulated in the transgenic lines, we performed RT-PCR to monitor the expression levels of cytosolic *APX* in fibres at four different developmental stages. The results of this analysis showed that only *GhAPX1A*_*T*_ */D*_*T*_ expression levels were down-regulated during all stages of development in IAU lines, while two *APXs* that were homologous to *Gorai.004G227100.1* and *Gorai.004G227300.3*, which had moderately high expression levels during the elongation stage (5–15 DPA), were also down-regulated in IAO lines (Fig. 3C).

To further confirm the expression levels in the transgenic lines, we evaluated the enzyme activities in 0 DPA ovules and 10 DPA fibres (see Supplementary Fig. S2 at *JXB* online). Cytosolic APX enzyme activities in the OA lines were up-regulated in 0 DPA ovules but remained unchanged in 10 DPA fibres. Cytosolic APX activities were decreased in both 0 DPA ovules and 10 DPA fibres in IAU lines, and the activities were completely inhibited in ovules and fibres in IAO lines (Supplementary Fig. S2A). In the IAO lines, down-regulation of *cAPX*s did not induce a complementary up-regulation of ROS scavenging enzyme activities such as SOD, CAT and POD (Supplementary Fig. S2). Taken together, these results implied that cytosolic APX expression levels and enzyme activities were significantly down-regulated in the IAO lines.

### Suppression of cytosolic *APX*s inhibited fibre development

**Fig. 4.**
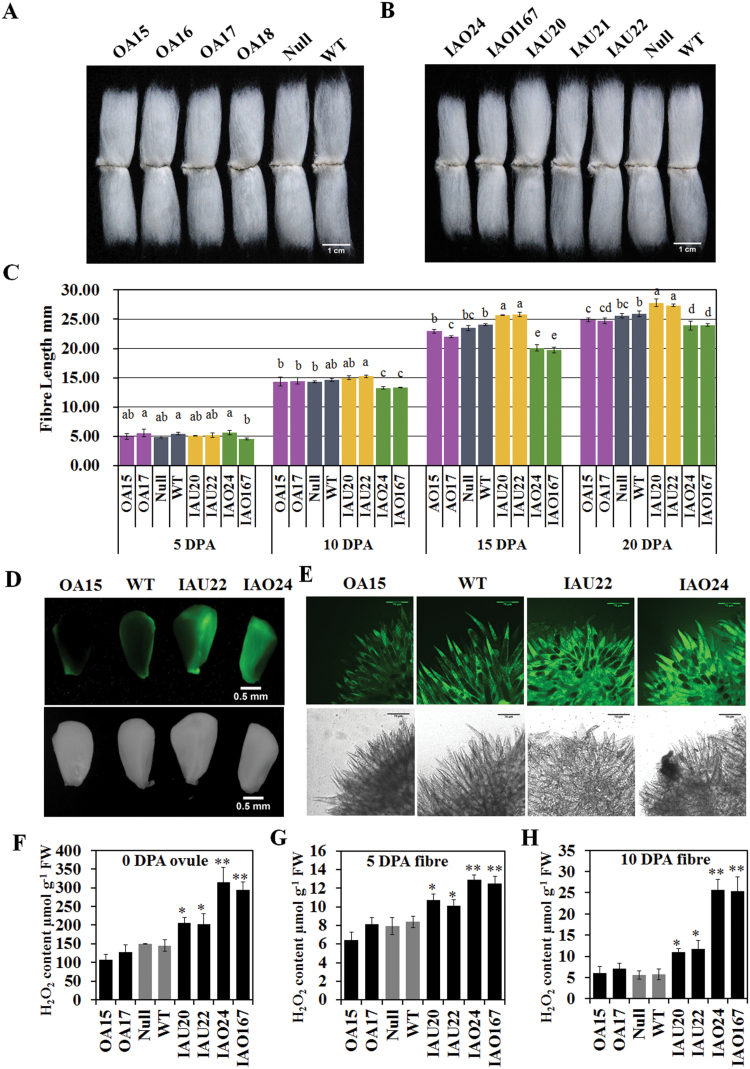
Fibre lengths and H_2_O_2_ (hydrogen peroxide) levels at different developmental stages of fibres in the transgenic lines. (A, B) Mature fibres of over-expression (A) and RNAi (B) lines compared with controls; bar=1cm. (C) Fibre lengths of transgenic lines during the fibre developmental stages of fibres (mean±SD, *n*=3). Values with different letters (a–e) indicate significant differences (Duncan’s multiple comparisons, *P*<0.05) at one stage. (D, E) Qualitative detection of H_2_O_2_ in 0 DPA ovules (D) and 2 DPA fibres (E) by staining with 10 µM 2′,7′-DCFDA and observing the results with a stereo fluorescence microscope and Laser TCS SP2 confocal spectral microscope, respectively. Scale bars: 0.5 µm (D); 150 µm (E). (F–H) Quantification of H_2_O_2_ in 0 DPA ovules (F), 5 DPA fibres (G) and 10 DPA fibres (H) by spectrophotometry (mean±SD, *n*=4). Asterisks indicate that the variance reached the level of significance compared with the null control (Student’s *t* test: *P<0.05, **P<0.01). OA15/16/17/18, individual over-expression lines; IAU20/21/22, individual *GhAPX1A*_*T*_ */D*_*T*_ RANi lines; IAO24/167, individual cytosolic *APXs* RNAi lines; Null, transgenic negative control; WT, wild type cotton YZ1.

To assess whether the modification of APX expression could affect fibre development, we measured the quality of the transgenic cotton fibres harvested from plants grown in the field over two years, the T3 generation in 2013 (see Supplementary Table S5 at *JXB* online) and the T4 generation in 2014 (Table 1), using High Volume Instrument (HVI) analysis. For the OA lines (T4), fibre lengths (27.25±0.42, 28.24±0.51, 27.88±0.49, and 26.89±0.67mm) decreased slightly compared with the null control (28.42±0.67mm) (Fig. 4A and Table 1). Specific suppression of *GhAPX1A*_*T*_ */D*_*T*_ (T4) slightly increased fibre length (28.79±0.35, 28.46±0.44, and 30.56±0.62mm) compared with the controls. However, suppression of whole cytosolic *APX* members (T4) in fibres significantly reduced the fibre length (26.06±0.31 and 25.71±0.63mm) by 8.30–9.54% (Fig. 4B and Table 1). The measurement of fibre lengths from 5 to 20 DPA confirmed the significant decrease in IAO fibre length. It also showed that 10 DPA was the key point at which the IAU and IAO lines first displayed different elongation rates (Fig. 4C) and the fibre lengths in the IAO lines were significantly reduced. For the IAO lines including the fibre length, all fibre quality indexes worsened, including the uniformity index, micronaire, elongation, maturity ratio and short fibre index, compared with the controls (Table 1 and Supplementary Table S5). Overall, down-regulation of cytosolic *APX* suppressed fibre development, including elongation and secondary cell wall synthesis.

**Table 1.** *Fibre quality analysis with a High Volume Instrument (HVI) harvested from T4 plants grown in fields (2014*) Mature fibres were harvested from experimental fields at Huazhong Agriculture University, Wuhan, Hubei province on one experimental plot. Five samples were collected from each line for the fibre quality measurements. Data are mean±SD, *n*=5. Values with different letters (a–f) have significant differences (Duncan’s multiple comparisons, *P*<0.05) in one fibre quality index. The two lines of which fibre quality is changed are shown in bold.

	UHML^*a*^ (mm)	Uniformity index (%)	Micronaire	Strength (g/tex)^*b*^	Elongation (%)	Maturity ratio	Short fibre index
OA15	27.25±0.42de	84.45±0.60cd	5.52±0.05b	25.74±0.63ab	6.79±0.07ab	0.85±0.005ab	7.89±0.51b
OA16	28.24±0.51bc	85.40±1.08bc	5.25±0.10c	24.85±0.88bcd	6.70±0.06bc	0.84±0.005c	7.20±0.36bcd
OA17	27.88±0.49cd	85.23±0.97bc	5.55±0.10b	26.08±1.29a	6.90±0.11a	0.85±0.006a	7.30±0.42bcd
OA18	26.89±0.67e	84.58±1.77bcd	5.84±0.07a	23.64±1.25e	6.52±0.08e	0.84±0.005abc	7.88±1.14b
Null	28.42±0.67bc	85.91±0.92ab	5.34±0.46bc	25.53±0.45abc	6.65±0.05c	0.84±0.012bc	7.02±0.25cd
WT	28.44±0.51bc	85.90±1.11ab	5.46±0.07bc	24.40±1.14cde	6.63±0.08cd	0.84±0.004bc	7.05±0.31cd
IAU20	28.79±0.35b	84.73±0.59bcd	5.43±0.09bc	25.27±0.98abc	6.66±0.05c	0.84±0.005abc	7.40±0.27bcd
IAU21	28.46±0.44bc	84.81±1.26bcd	5.50±0.10b	23.92±0.66de	6.63±0.08cd	0.84±0.000bc	7.48±0.53bc
IAU22	30.56±0.62a	86.86±0.60a	5.39±0.07bc	26.05±0.84a	6.85±0.05a	0.84±0.005abc	6.72±0.04d
**IAO24**	**26.06±0.31f**	**83.70±0.86de**	**3.62±0.20d**	**24.85±0.26bcd**	**6.55±0.06de**	**0.80±0.010d**	**8.45±0.67a**
**IAO167**	**25.71±0.63f**	**83.08±0.94e**	**3.66±0.13d**	**23.70±0.32e**	**6.23±0.13f**	**0.79±0.000d**	**8.97±0.82a**

^*a*^ UHML: upper half mean length; the mean length of the top half of the fibres.

^*b*^ Values reported as grams per tex, which is the force in grams required to break a bundle of fibres 1 tex unit in size, where a tex unit is equal to the weight in grams of 1000 m of fibre.

### Increased H_2_O_2_ levels during fibre elongation were coupled to suppressed fibre elongation

APX uses ASA as the electron donor to reduce H_2_O_2_ to H_2_O, while ASA is oxidized to DHA (Foyer and Noctor, 2011). We measured H_2_O_2_ and ASA contents in ovules and fibres at different stages. Fluorescence detection of ROS using 2′,7′-DCFDA in 0 DPA ovules and 2 DPA fibres demonstrated that ROS levels were unchanged in the overexpression (OA) lines but increased in the RNAi (IAU and IAO) lines (Fig. 4D, E). Quantitative determination of H_2_O_2_ concentrations also revealed no significant difference between OA lines and controls in 0 DPA ovules, 5 DPA fibres, or 10 DPA fibres (Fig. 4F–H). In the IAU and IAO lines, the H_2_O_2_ levels increased in both ovules and fibres, and the increases in the IAO lines were more dramatic than those in the IAU lines. In 10 DPA fibres, the H_2_O_2_ content in the IAO lines was approximately 4.5-fold higher than that in the controls, and double the levels were detected in the IAU lines compared with the controls (Fig. 4H). Additionally, the ASA contents and ASA/DHA ratios were significantly increased in IAO lines compared with the controls (see Supplementary Fig. S3 at *JXB* online). These results suggested that the serious increase in H_2_O_2_ levels altered the cellular redox status of fibres in IAO, which might reduce fibre elongation.

### Down-regulation of the *cAPX* family reduced fibre elongation by inducing oxidative stress

To determine how the increase of H_2_O_2_ in cytosolic APX suppression lines reduced the fibre elongation, we performed RNA-seq analysis to identify differentially expressed genes in the control and RNAi lines in 10 DPA fibres.

Total RNA were extracted from 10 DPA fibres of Null, wild type (WT), IAU20, IAU22, IAO24 and IAO167 lines for RNA-seq (see Supplementary Fig. S4 at *JXB* online). Six samples were divided into three groups. The Null and wild type (WT) represented the control group, IAU20 and IAU22 designated the IAU group, and IAO24 and IAO167 were the IAO group. Overall, 184 differentially expressed genes (DEGs) were screened out by group comparison using the NOISeq method (Supplementary Fig. S5 and Supplementary Table S6 at *JXB* online), and 21.21% of the genes had an unknown function. There were 126 DEGs identified in IAO compared with the control, 17 DEGs in IAU compared with the control, and 134 DEGs in IAO compared with IAU (Supplementary Fig. S5). According to the RNA-seq results, only *GhAPX1A*_*T*_ */D*_*T*_ were silenced in IAU lines and the expression levels of other *APX*s remained unchanged. However, all *cAPX*s were decreased in IAO (Supplementary Fig. S4B), which was consistent with the RT-PCR results (Fig. 3C).

Several genes related to oxidative stress were up-regulated in the IAO RNAi lines, such as redox homeostasis related genes (12.63% of DEGs), ribosomal proteins (7.07% of DEGs), proteases and heat-shock proteins (sHSP20, HSP70) (9.60% of DEGs) for protein maintenance (see Supplementary Figs S5B and S6). These results suggested that other stress response genes were induced to resist the oxidative stress caused by the suppression of cytosolic APX.

### Cytosolic *APX*s were the key factors for the maintenance of fibre tolerance to oxidative stress

**Fig. 5.**
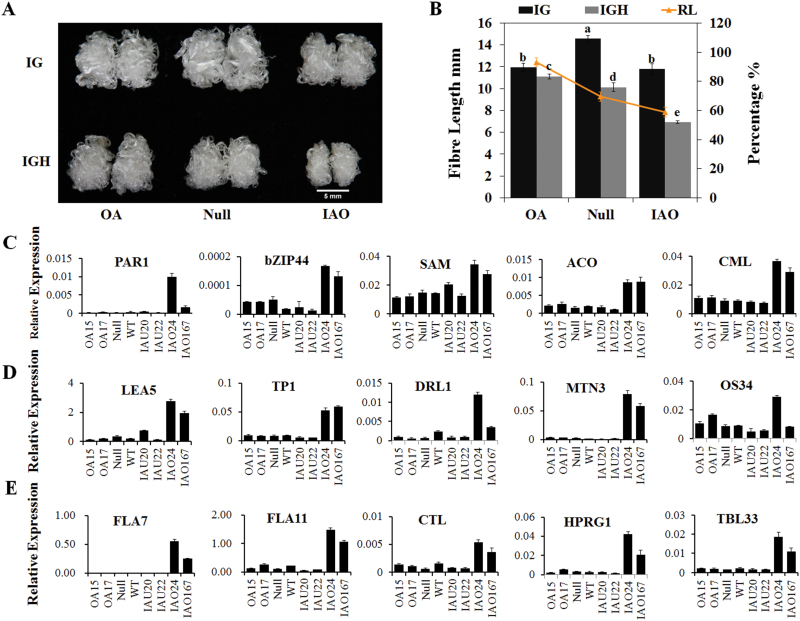
Sensitivity of transgenic cotton to oxidative stress and RT-qPCR validation of differentially expressed genes (DEGs). (A) Fibres in the OA line were less sensitive to oxidative stress in the presence of 1mM H_2_O_2_ in ovule culture medium. IG: 5 µM IAA and 0.5 µM GA_3_; IGH: 5 µM IAA, 0.5 µM GA_3_ and 1mM H_2_O_2_. Scale bar: 5mm. (B) The fibre length analysis was performed after culturing the ovules with 1mM H_2_O_2_. RL indicates the fibre length percentage in response to treatments compared with non-treatment for each line (mean±sd, n=8).) Values with different letters (a, b, c and d) above the bars in the histogram indicate significant differences (Duncan’s multiple comparisons, *P*<0.05). (C-E) RT-qPCR verification of DEGs related to signal transduction (C), stress response (D) and secondary cell wall synthesis genes (E) in 10 DPA fibres.

Although *GhAPX1A*_*T*_ */D*_*T*_ expression levels did not increase during the rapid elongation stage in OA lines, the gene transcripts were relatively more abundant in 0 DPA ovules and 15 DPA fibres compared with the controls (Fig. 3C). Next, we questioned whether the up-regulation of *GhAPX1A*_*T*_ */D*_*T*_ in OA lines improved the tolerance of fibres to oxidative stress, which might be predicted based on the expected protein function. We cultured transgenic OA, IAO and null ovules under standard culture conditions and oxidative stress conditions in the presence of 1mM H_2_O_2_. After 10 days of culture with 1mM H_2_O_2_, the fibre length in the OA samples decreased only 6.84% compared with the fibres under unstressed conditions (11.11mm compared with 11.94mm) (Fig. 5A, B), whereas the null controls decreased 30.44% compared with the unstressed conditions (10.12mm compared with 14.56mm). The fibre lengths in the IAO lines were reduced 41.26% (6.91mm compared with 11.79mm) (Fig. 5A, B). These results were consistent with the speculation that overexpression of *GhAPX1A*_*T*_ */D*_*T*_ reduced the sensitivity of fibres to high levels of oxidative stress.

These results demonstrated that *GhAPX1A*_*T*_ */D*_*T*_ over-expression increased the resistance to oxidative stress in the presence of 1mM H_2_O_2_, whereas lines with suppressed cytosolic *APX*s were more sensitive to oxidative stress compared with the control. These findings confirmed that cytosolic APXs maintained the cellular redox status for fibre elongation to avoid oxidative stress during the rapid elongation stage of the fibre.

### Earlier expression of secondary cell wall-related genes during the elongation stage correlated with shorter fibres in the IAO lines

**Fig. 6.**
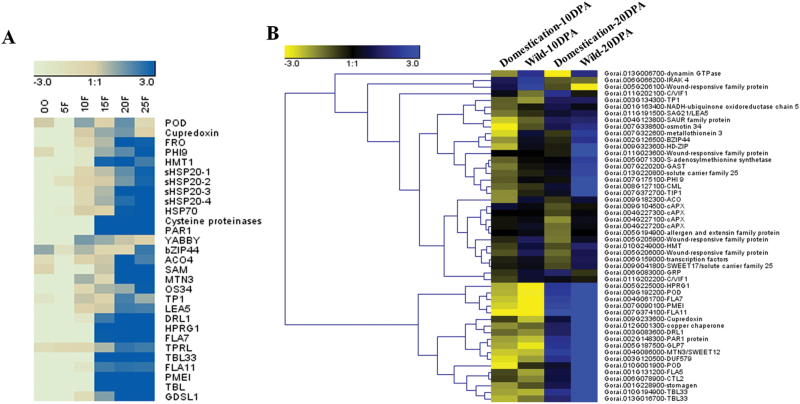
Expression profile analysis of DGEs (differentially expressed genes) in 10 DPA and 20 DPA fibres of wild and domesticated cotton. (A) Heat map of the expression levels of DEGs during the developmental stages of YZ1 fibres. Cluster analysis was performed with relative expression levels that were normalized to the expression of the cotton *GhUB7* gene using RT-qPCR. (B) DEGs between IAO lines and control lines revealed different expression levels between domesticated cotton and wild cotton. The mean RPKMs of five domesticated cottons and four wild cottons (SRA061240) were used to construct the heat map.

Chaudhary *et al.* (2009) proposed that the longer fibre of domesticated cotton was the result of an enhanced ability to modulate the cellular redox state, resulting in a delay in the onset of the effects of stress. For our RNA-seq results, the majority of the DEGs were confirmed by RT-qPCR analysis in the transgenic lines and controls (Fig. 5C–E). We also found that the DEGs were classified into several clusters, such as signal transduction (*PAR1*, *bZIP44*, *SAM* (*S-adenosyl methionine synthetase*), *ACO* and *CML*) (Fig. 5C), stress response (*Late Embryogenesis Abundant Protein 5* (*LEA5*), *Pathogenesis Related Thaumatin Superfamily Protein* (*TP1*) and *Dirigent-Like Protein* (*DRL1*)), osmotic pressure-related (*MTN3/SWEET12* and *OS34* (*OSMOTIN 34*)) (Fig. 5D) and cell wall synthesis-related (*FLA Fasciclin-Like Arabinoogalactan 7/11* (*FLA7/11*), *Chitinase-Like (CTL*), *Hydroxyproline-Rich Glycoprotein* (*HPRG1*), and *Trichome Birefringence-Like* (*TBL/TBL33*)) (Fig. 5E). Moreover, most of the up-regulated genes in 10 DPA fibres of the IAO lines were related to secondary cell wall synthesis and should be preferentially expressed in the secondary cell wall synthesis stage of WT plants (after approximately 15 DPA) (Fig. 6A), but were initiated early in the fibres of IAO lines.

Therefore we hypothesized that these DEGs might display different expression levels in domesticated and wild cotton with different fibre lengths. To explore this possibility, the raw data (SRA061240) were downloaded from NCBI. Next, we mapped these reads to the *G. raimondii* genome to calculate the RPKM values of the genes. The expression profiles of the DEGs were analysed in 10 DPA and 20 DPA fibres of five domesticated (TM-1, Maxxa, CRB252, Coker315, CascotL7) and four wild *G. hirsutum* varieties (yucTX2090, yucTX2094, yucTX2095, palmeriTX665) (Fig. 6B). Most of the DEGs displayed higher expression levels in fibres of wild cotton than domesticated cotton at each stage (10 or 20 DPA). In wild cotton fibres at 10 DPA, the up-regulated genes (compared with domesticated cotton) were associated with signal transduction, stress responses, such as *bZIP44*, *HD-ZIP*, *SAM*, *ACO*, and *LEA5*, and wound response family proteins, among others. In 20 DPA fibres, up-regulated genes were largely associated with cell wall processes and stress responses, such as *FLA7/11*, *CTL*, *TBL*, *HPRG1*, *MTN3*, and *DRL1*, among others (Fig. 6B). Therefore, our results provided evidence to support the hypothesis that the longer fibre of domesticated cotton resulted from the improved anti-oxidative capacity (Hovav *et al.*, 2008).

## Discussion

### A previously unrealized group of cAPXs contributed to redox equilibrium control in elongating fibre cells

APX and CAT are the two major enzymes that scavenge H_2_O_2_ in plant cells to maintain redox homeostasis. APX may be involved in controlling H_2_O_2_ content for signalling, whereas CAT is mainly involved in H_2_O_2_ removal as part of the stress response (Mittler, 2002). By mining the available public data together with our results, we found that cytosolic *APX*s were up-regulated in 10 DPA fibres of domesticated upland cotton compared with wild cotton, especially cytosolic *GhAPX1A*_*T*_ */D*_*T*_ (see Supplementary Tables S3, S7 and S8 at *JXB* online). More cytosolic *APX* transcripts that accumulated during the domesticated fibre elongation stage should improve redox homeostasis during rapid fibre elongation.

To evaluate the evolution of cAPX proteins in plants, all cAPX sequences from sequenced species were aligned and proteins were divided into several clades depending on the sequence similarity (see Supplementary Fig. S7 at *JXB* online). The cAPXs from *G. raimondii* and *G. arboreum* and a cAPX from *Theobroma cacao* constituted a previously unrealized group of cAPXs in dicotyledonous plants, which showed a relatively high sequence similarity to cAPXs from monocotyledonous plants (Supplementary Fig. S7). The expansion of cAPX members in *Gossypium* compared with other species has been discovered. There were six cAPXs in diploid and 12 cAPXs in allotetraploid cotton, illustrating the amplification of the gene family in *Gossypium* (Supplementary Table S9 at *JXB* online). Family expansion had likely arisen by gene duplication. For example, *Gorai.004G227100.1*, *Gorai.004G227200.2*, and *Gorai.004G227300.3* in *G. raimondii* (D5) and *Cotton_A_05101*, *Cotton_A_05102*, and *Cotton_A_05103* in *G. arboretum* (A2) were closely linked on one chromosome and showed very high sequence similarity to one another. The process also appeared to have occurred in *G. hirsutum*. *GhAPX1A*_*T*_ */D*_*T*_ showed a high sequence similarity with the expanded family members, and together they formed the previously undescribed cAPX group (Supplementary Fig. S7). We hypothesized that the expansion of *cAPX*s in the *Gossypium* genome led to an improved ability to control fibre cell redox state equilibrium.

### Oxidative stress resulted in short fibres by initiating secondary cell wall-related gene expression

According to a previous report, the long fibre was correlated with stronger ROS scavenging ability than the short fibre (Hovav *et al.*, 2008). The transcriptome comparison between domesticated and wild cotton fibres also suggested that breeding may have selected for an enhanced capacity for cellular redox state modulation in domesticated cotton fibre initiation and elongation (Hovav *et al.*, 2008; Chaudhary *et al.*, 2009; Yoo and Wendel, 2014). Thus, it was important to correctly control redox homeostasis during the different stages of fibre development. That the suppression of all cytosolic *APX*s significantly increased fibre ROS levels and decreased fibre length in our research also supported this hypothesis.

Cotton fibre length is determined by the duration period of fibre elongation, and fibre elongation is terminated or slowed down by the activation of secondary cell wall synthesis. H_2_O_2_ can activate cellulose synthase (CesA) dimerization, promoting the synthesis of cellulose and triggering secondary cell wall formation, including phenylpropanoid biosynthesis and lignin biosynthesis (Kurek *et al.*, 2002; Han *et al.*, 2013). Previous reports have shown there were very low ROS levels in young cotton bolls, but a dramatic increase has been observed at 15–20 DPA, followed by a drop in 25 or 26 DPA fibres (Potikha *et al.*, 1999; Yang *et al.*, 2008). At approximately 15 DPA, fibre cells initiate the transition to secondary cell wall synthesis, which was confirmed by the birefringence of fibres and the direct quantitative analysis of cellulose content (Potikha *et al.*, 1999; Stiff and Haigler, 2013). The production of H_2_O_2_, which was uniformly distributed at the cell surface, was first seen using dihydrorhodamine fluorescence in 15–18 DPA fibres, and exogenous application of H_2_O_2_ to younger fibres caused a premature initiation of secondary wall deposition (Potikha *et al.*, 1999). In the IAO lines, many genes related to secondary cell wall synthesis were up-regulated at 10 DPA, potentially due to the high accumulation of H_2_O_2_. Therefore, we prefer the hypothesis that high H_2_O_2_ concentrations act as a signalling event for the initiation of secondary cell wall thickening.

The analysis of DEGs confirmed that oxidative stress in the RNAi lines resulted in relatively short fibres by initiating cell wall-related gene expression. These DEGs included *FLA7/11*, *Hydroxyproline-Rich Glycoprotein* (*HPRG1*), and *Trichome Birefringence-Like 33* (*TBL33*), among others. *FLA7* and *FLA11* are expressed during the secondary cell wall stage (Yoo and Wendel, 2014). *HPRG1* represents a large family of cell wall extension proteins that are involved in cross-linking and increasing the strength and toughness of the primary cell wall (Hijazi *et al.*, 2014). *TBL/TBL33* genes are expressed during the secondary cell wall stage and involved in O-acetylation of cell wall polysaccharides. O-Acetyl groups on plant polysaccharides significantly increase the cell wall viscosity and gelation properties, and they decrease the efficiency of the enzymatic degradation of pectin and xylan. Therefore, acetylation might constitute a barrier to cell wall deconstruction (Gille and Pauly, 2012). The up-regulation of cell wall structures and modifying proteins in the IAO RNAi lines suggested that oxidative stress-induced cell wall modification might restrict fibre elongation.

The earlier expression of secondary cell wall-related genes during the elongation stage might be mediated by some signal transduction genes. Several DEGs screened from the IAO RNAi lines were hypothesized to participate in signalling pathways to regulate fibre development and initiate secondary cell wall synthesis. For example, the Ca^2+^ receptor CML and ethylene synthesis enzyme genes SAM and ACO were induced in IAO fibres (Fig. 5C). Although the function of CML in fibre development has not been described, we have previously reported Ca^2+^ signalling related to *GhCaM7*. Over-expression of the calmodulin gene *GhCaM7* was found to induce H_2_O_2_ accumulation in fibres, promoting fibre elongation during the early fibre elongation stage and inhibiting fibre elongation during later stages (Tang *et al.*, 2014). ETH-regulated fibre development depended on the ETH content during the elongation stage; higher or lower levels inhibited elongation, and intermediate levels promoted fibre extension (Shi *et al.*, 2006; Li *et al.*, 2014). Therefore, an increase in endogenous H_2_O_2_ levels likely induced abnormal Ca^2+^ and ethylene signalling, resulting in reduced fibre elongation in the IAO RNAi lines.

The complexity of the cotton genome as a result of polyploidization appeared to contribute to the enhanced adaptability to the environment and led to a high degree of plasticity of ROS signalling in fibre development. Domestication further improved the cellular redox state modulation capacity. ROS and other signalling events together control fibre development. Due to the complicated crosstalk, it will be a challenge to improve fibre quality by regulating the expression of one or two genes related to ROS. Techniques to regulate ROS signalling to improve fibre quality using genetic engineering approaches require further study.

## Supplementary data

Supplementary data are available at *JXB* online.

Figure S1. Southern blotting of transgenic plants.

Figure S2. Four types of enzymatic activities detected in 0 DPA ovules and 10 DPA fibres of transgenic lines.

Figure S3. Detection of ASA (ascorbic acid) and DHA (dehydroascorbic acid) contents in transgenic lines in 5 DPA fibres.

Figure S4. DEGs (differentially expressed genes) between each two samples.

Figure S5. DEGs (differentially expressed genes) compared across groups.

Figure S6. RT-qPCR of protein maintenance and oxidative stress-related DEGs (differentially expressed genes) in 10 DPA fibres of transgenic lines.

Figure S7. Phylogenetic analysis of cytosolic APX from bryophytes, lichens, monocots and dicots.

Table S1. List of primers used in this study.

Table S2. Lists of total reads mapped to the *Gossypium raimondii* genes and genome.

Table S3. RPKM values of APXs in different fibre stages of *G. hirsutum* TM-1.

Table S4. List of all transgenic lines in the different generations.

Table S5. Fibre quality analysis with HVI (High Volume Instrument) harvested from T3 plants grown in fields (2013).

Table S6. List of DEGs (differentially expressed genes) screened by group comparison of the transgenic lines.

Table S7. RPKM values of APX family members in 10 DPA and 20 DPA fibres of domesticated and wild *G. hirsutum*.

Table S8. RPKM values of APXs in different fibre stages of *G. raimondii*.

Table S9. Classification of APX genes in different species according to the amino acid sequences.

Supplementary Data
