# Supplementary material for: Fibre elongation requires normal redox homeostasis modulated by cytosolic ascorbate peroxidase in cotton (Gossypium hirsutum)
Source: J Exp Bot. 2016 Apr 17;67(11):3289–301. doi: 10.1093/jxb/erw146 (PMC4892722; doi:10.1093/jxb/erw146)
Supplement: Supplementary Data [file supp_67_11_3289__index.html]

Fibre elongation requires normal redox homeostasis modulated by cytosolic ascorbate peroxidase in cotton (Gossypium hirsutum) — Fibre elongation requires normal redox homeostasis modulated by cytosolic ascorbate peroxidase in cotton (Gossypium hirsutum) — Supplementary Data 

# Fibre elongation requires normal redox homeostasis modulated by cytosolic ascorbate peroxidase in cotton (*Gossypium hirsutum*)

## Supplementary Data

Data files

- supplementary\_figures\_S1\_S7\_tables\_S1\_S9.pdf - Supplementary Data
