# Supplementary material for: Fibre elongation requires normal redox homeostasis modulated by cytosolic ascorbate peroxidase in cotton (Gossypium hirsutum)
Source: J Exp Bot. 2016 Apr 17;67(11):3289–301. doi: 10.1093/jxb/erw146 (PMC4892722; doi:10.1093/jxb/erw146)
Supplement: Supplementary Data [file supp_erw146_supplementary_figures_S1_S7_tables_S1_S9.pdf]

**The *Journal of Experimental Botany* Supplementary Data**

Article title: Fibre elongation requires normal redox homeostasis modulated by cytosolic ascorbate peroxidase (cAPX) in cotton (*Gossypium. hirsitum*)

Authors: Kai Guo, Xueqiong Du, Lili Tu*, Wenxin Tang, Pengcheng Wang, Maojun Wang, Zhen Liu, Xianlong Zhang

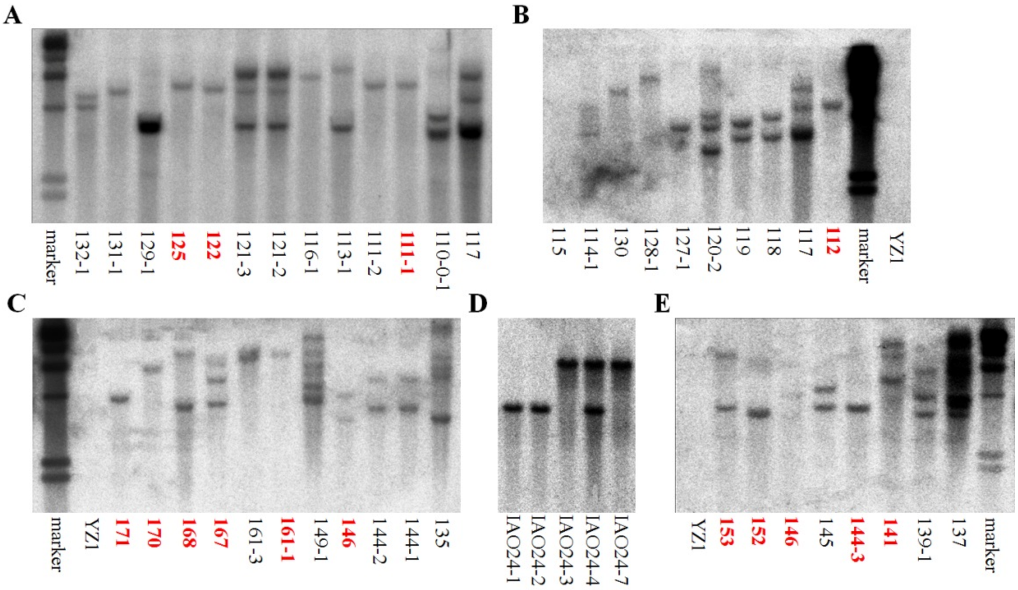


**Supplementary Fig. S1.** Southern blotting of transgenic plants. (A-B) Southern blotting of 35S over-expression lines. (C) Southern blotting of IAO lines. (D) A single plant from the IAO24 line was analysed by Southern blotting. IAO24-2 and IAO24-3 were different transformants that were separated from the same T0 plant (168/IAO24). (E) Southern blotting of IAU lines. *NPT II* was used as the probe. λ DNA digested with *Hind*III was used as the marker.

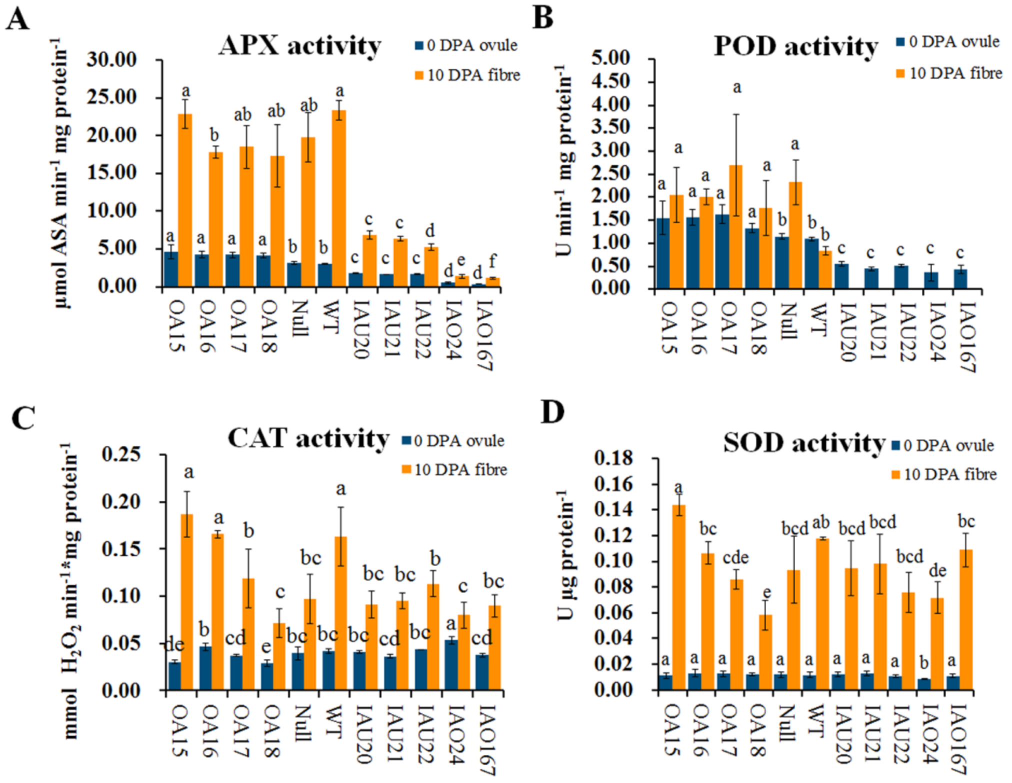


**Supplementary Fig. S2**. The activities of four types of enzymes were detected in 0 DPA ovules and 10 DPA fibres of the transgenic lines. (A), APX (ascorbate peroxidase) enzyme activity. (B), POD (type III peroxidase) enzyme activity. (C), CAT (catalase) enzyme activity. (D), SOD (superoxide dismutase) enzyme activity. Values with different letters (a, b, c d and e) above the bars in the histogram indicate significant differences at one development stage. (Mean ± se, n=3) (Duncan's multiple comparisons, $P < 0.05$).

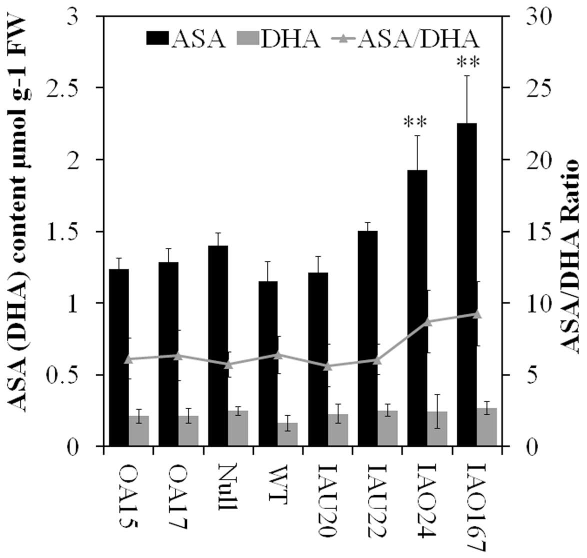


**Supplementary Fig. S3**. ASA (ascorbic acid) and DHA (dehydroascorbic acid) content in transgenic lines in 5 DPA fibres. *Indicates that the variance was very significant at $P$ < 0.01 (**) compared with the null control. (mean ± sd, n=4), (Student's $t$ test).

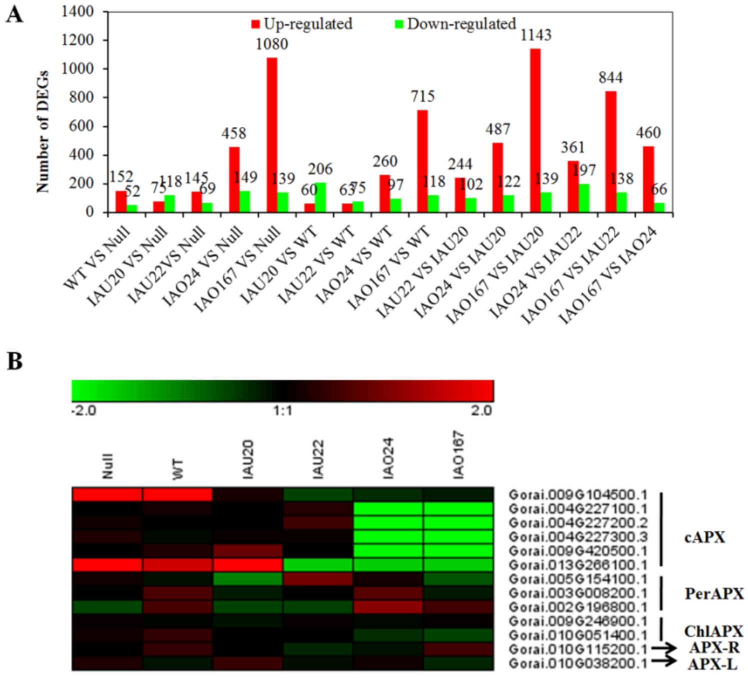


**Supplementary Fig. S4**. DEGs (Differentially Expressed Genes) between each two samples. (A) The number of DEG was compared between two samples. The filtering criteria were log2 ⩾ 1 and RPKM ⩾ 0.1. (B) Expression profiles of the APX family members in six samples.

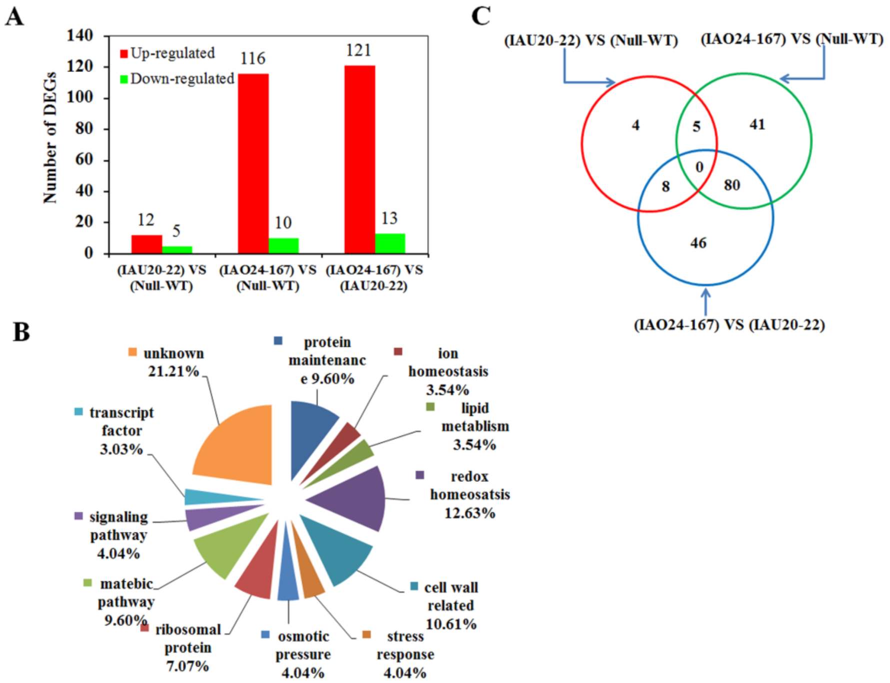


**Supplementary Fig. S5**. DEGs (Differentially Expressed Genes) compared across groups. (A) Numbers of DEGs in the group comparison. (B) Gene cluster analysis. (C) The distribution of DEGs across three groups. Null and WT made up the Null-WT control group. IAU20 and IAU22 made up the IAU group. IAO24 and IAO167 made up the IAO group.

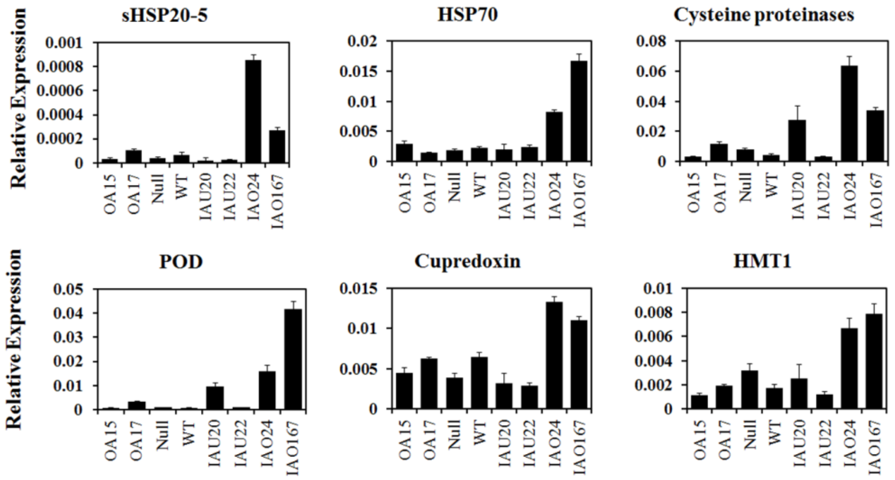


**Supplementary Fig. S6**. RT-qPCR of protein maintenance and oxidative stress-related DEGs (Differentially Expressed Genes) in 10 DPA fibres of transgenic lines. Relative expression levels were normalised against the expression of the cotton *GhUB7* gene.

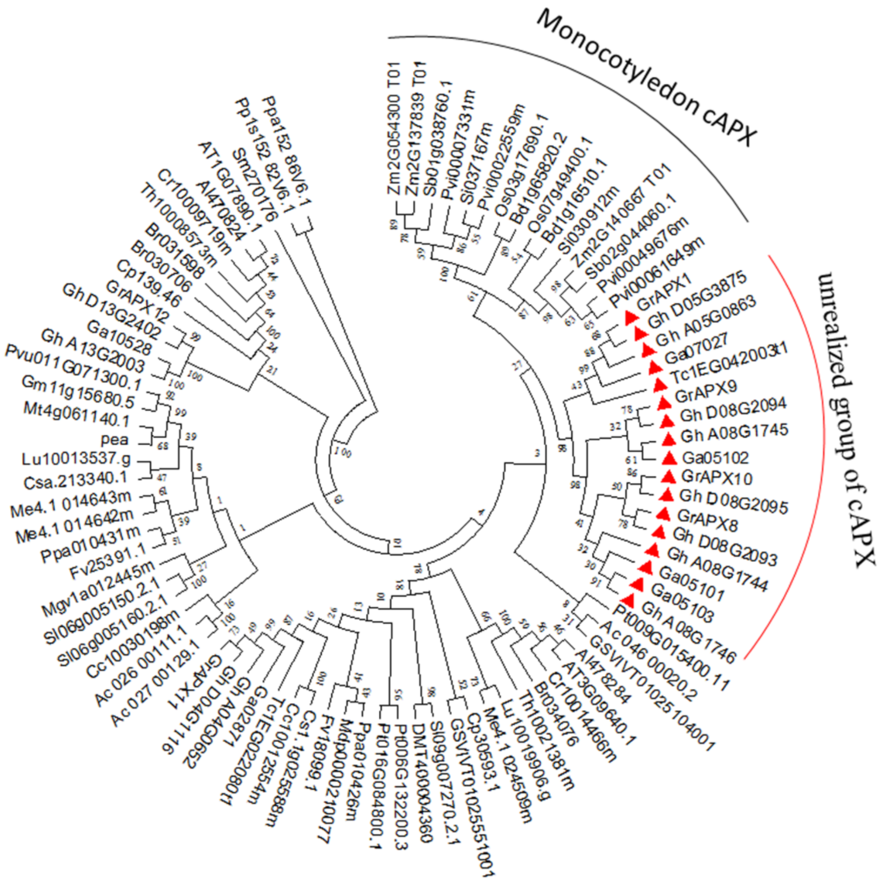


**Supplementary Fig. S7**. Phylogenetic analysis of cytosolic APX from bryophytes, lichens, monocots and dicots. The tree was constructed using the neighbour-joining method with pairwise deletion. Amino acid sequences were used to calculate molecular distances. The red triangles indicate the unrealised group of the cytosolic APXs from dicots. All protein sequences were downloaded from Phytozome 9.1 (http://www.phytozome.net/cotton.php). A phylogenetic tree was constructed with MEGA 5.1. Molecular distances were calculated using the p-distance parameter. The phylogeny test employed the bootstrap method with 1000 replications.

**Supplementary Table S1**. List of primers used in this study.

| primer name | primer sequence | used for |
|---|---|---|
| GhAPX1A$_T$ORFsens | ATGACCAAGTGTTACCCAACTGTG | RT-PCR/Northe |
| GhAPX1A$_T$ORFanti | TTATGCATCAGCAAATCCTAGCTC | rn |
| GhAPXA$_T$ORF-anti | GGGGACCACTTTGTACAAGAAAGCTGG GTcTTATGCATCAGCAAATCCTAGCTC | 35s-GhAPX1/ORF-RNAi |
| GhAPXA$_T$ORF-sens | GGGGACAAGTTTGTACAAAAAAGCAGG CTtgATGACCAAGTGTTACCCAACTGTG | 35s-GhAPX1/ORF-RNAi |
| GhAPX1A$_T$-3UTR-sens | GGGGACAAGTTTGTACAAAAAAGCAGG CTtgGCTGATGGTTGCGGAATAG | 3'UTR-RNAi |
| GhAPX1A$_T$-3UTR-anti | GGGGACCACTTTGTACAAGAAAGCTGG GTcCAAACTCAATTCAACATGTTCAAC | |
| GhAPX1A$_T$3GSP1 | ATGACCAAGTGTTACCCAACTGTGAGCG | 5'RACE |
| GhAPX1A$_T$3GSP2 | GCTTGCTGGTGTCGTTGCCGTTGA | |
| GhAPX1A$_T$5GSP1 | AGAAGGCATCCTCATCGGCAGCATA | 3'RACE |
| GhAPX1A$_T$5GSP2 | CTCCTTGTGGCACCTTCCCAGAGTG | |
| GhAPX1A$_T$/D$_T$-RT-S2 | GCCGTTGAGATTACTGGTGGA | realtime PCR |
| GhAPX1A$_T$/D$_T$-RT-AS2 | GACCCATTTGAGCACTAAACACC | |
| GhUB7S | GAAGGCATTCCACCTGACCAAC | realtime PCR |
| GhUB7AS | CTTGACCTTCTTCTTCTTGTGCTTG | |
| 18S RNAS | GCTCGTAGTTGGACTTAGGGTGGGT | Northern |
| 18S RNAAS | GGTCGGCATCGTTTATGGTTG | |
| Gorai.004G227100.1RTS1 | CGTGGCTTTCCTATCTTCATTTC | realtime PCR |
| Gorai.004G227100.1RTAs2 | TCCTCGCTAACGGTTGGGTAA | |
| Gorai.004G227300.3RTS2 | GCCAACGACACACAGATGTTTTAG | realtime PCR |
| Gorai.004G227300.3RTAS2 | AACGGATAACAAGTTTCAACACAA | |
| CTLS | CTTCTACACCAAGATTCCCCCTAT | realtime PCR |
| CTLAS | CCAATGGCTTCACTGATTCCTG | |
| CMLS | AACAAGGGAAGAACAACAACGAA | realtime PCR |
| CMLAS | TCTTCAAACTCTCGGCTGTTATCA | |
| PMEI-S | TTCAGAAACACCGCTGGTCCTAC | realtime PCR |
| PMEI-AS | CGGTCCCGTAGATGTCACATTCT | |
| FLA11-S | GATAAGGTGCTTTTGCCGTTGGA | realtime PCR |
| FLA11-AS | AATCAAAATCCCCCCAAAATAGCC | |
| TBL33-S | AAGCTCGACTCTCTCCTTACCTCT | realtime PCR |
| TBL33-AS | ATCCTTCTTCGCCCTTCCCTAT | |
| TBL-S | GCCGAGCTATCTCTAGACCAGTGA | realtime PCR |
| TBL-AS | GATTCTTCATACAGCGGACGACTC | |
| HMT1-S | TTTGAGACGAAAGATGGGAGTAGA | realtime PCR |
| HMT1-AS | ACCTTACGAGCACAACCTTCACA | |
| PHI9-S | CTACAACAATGGTGGTGAAGGTG | realtime PCR |
| PHI9-AS | TTTAAGCAAATCAACAGGGACAG | |
| FRO-S | TGACTCCTGATGGACCTCAAGAAT | realtime PCR |
| FRO-AS | AACACCAGCACAGGTAGAGCAAG | |

| | | |
|---|---|---|
| Cupredoxin-S | TCACTGTGCGAATGGAATGAAAC | realtime PCR |
| Cupredoxin-AS | ACAACAGCCATAAATGGGGAGAG | |
| POD-S | GTTGGGTAATCCTAAAATGGGTTC | realtime PCR |
| POD-AS | ATAGCAGAGGTGACAGTGGATTTC | |
| SHSP1-S | ATTACCCAGACAAGCAAAGCAGA | realtime PCR |
| SHSP1-AS | ACGTCAAGAGAGAATGGATCAAAG | |
| SHSP2-S | GGCAACAGACGCAGCAACAAC | realtime PCR |
| SHSP2-AS | GTTTCCCTCCAGTCAATCCGA | |
| SHSP3-S | ACATTGTTCAGTCGGGTGGTATT | realtime PCR |
| SHSP3-AS | GCATCACCATTTCATCATACTTCA | |
| SHSP4-S | ATCATTGTTTCGGTTGGGTAAGC | realtime PCR |
| SHSP4-AS | TTTCTGGGGAGCCTGTCGTCA | |
| SHSP5-S | GCACTGGCTCGTTTGGTTTTG | realtime PCR |
| SHSP5-AS | CCATTCCACCTCTGCCTTTGC | |
| SHSP6S | ACCTGAACCTAAGAAGCCCAAGA | realtime PCR |
| SHSP6AS | AACTCACACAACCAGAATGAAACAC | |
| HSP70-S | TCCCGCAAATCACCGTGTGTT | realtime PCR |
| HSP70-AS | TTTCGCCTCAACTTTCTTCTTATGT | |
| Cysteine proteinases-S | AATGCCGTGTTATCAGTTGTGGT | realtime PCR |
| Cysteine proteinases-AS | AAGGCTTGTTGTCATTGTTGGTG | |
| DRL1-S | CTACTTTGATGACTGATGCCTTTG | realtime PCR |
| DRL1-AS | ATTACAGTCTCATACGGTGCTTCTT | |
| TP1-S | ATGCTCAGCCACCTGCTCTTACT | realtime PCR |
| TP1-AS | AAGGAGCGGTGGGTCAGTGTT | |
| LEA5-S | CGTCGGAGACAAGGGCTTATTC | realtime PCR |
| LEA5-AS | GCGGTGGCAAGTTTCTACTGTG | |
| MTN3-S | ACAACAAGGTCCAATTAGAGCAAT | realtime PCR |
| MTN3-AS | ACTTGTGAGTTGCTAATCATCCCT | |
| OS34-S | CAGTGCCCTAATCAGTTGAAAGC | realtime PCR |
| OS34-AS | AGAACCTGGACAAATCCGTTGG | |
| SAM-S | AGATTTCACTTGGGAGGTTGTCA | realtime PCR |
| SAM-AS | TTCTCATTGCTATTTCATTCATTACC | |
| ACO-S | CATTGCTTCCTTCTACAACCCG | realtime PCR |
| ACO-AS | GCCAGCATAGAGCTTCATGTAGTC | |
| BZIP44-S | ATCTTTCTCAGTTGTCTTCCTCCAC | realtime PCR |
| BZIP44-AS | TCTTCTTGCTGATTCCCTGTTTG | |
| PAR1-S | TGCGAAGATTTGCCCAACGAT | realtime PCR |
| PAR1-AS | AAGAAGGGAATCGGATGAAATGC | |
| YABBY-S | AGCAAGGAGTAGAGTTACGGGAATA | realtime PCR |
| YABBY-AS | GTTATAGCAACCATACCACAAGCG | |
| FLA7-S | ATCCGAAGAAACTGGTTCCTCAT | realtime PCR |
| FLA7-AS | CCATCCACCTAAGAATGCCAA | |
| HPRG1-S | TAGCATTCGTCACCCTTTCTTCC | realtime PCR |
| HPRG1-AS | GCATTTCAGGCTTCTCAAGTTTT | |

**Supplementary Table S2**. Lists of total reads mapped to *Gossypium raimondii* genes and genome.

The list of total reads mapped to *G. raimondii* Gene

| Sample ID | Null | WT | IAU20 | IAU22 | IAO24 | IAO167 |
|---|---|---|---|---|---|---|
| Total Reads | 12434248(100%) | 11719314(100%) | 12329128(100%) | 12200881(100%) | 12435938(100%) | 12377822(100%) |
| Total BasePairs | 609278152(100%) | 574246386(100%) | 604127272(100%) | 597843169(100%) | 609360962(100%) | 606513278(100%) |
| Total Mapped Reads | 10419035(83.79%) | 9722960(82.97%) | 10209530(82.81%) | 10142320(83.13%) | 10196148(81.99%) | 10099130(81.59%) |
| perfect match | 6017861(48.4%) | 5554469(47.4%) | 5890185(47.77%) | 5828836(47.77%) | 5869132(47.19%) | 5831075(47.11%) |
| <=2bp mismatch | 4401174(35.4%) | 4168491(35.57%) | 4319345(35.03%) | 4313484(35.35%) | 4327016(34.79%) | 4268055(34.48%) |
| unique match | 10003362(80.45%) | 9342655(79.72%) | 9816695(79.62%) | 9741754(79.84%) | 9778887(78.63%) | 9685212(78.25%) |
| multi-position match | 415673(3.34%) | 380305(3.25%) | 392835(3.19%) | 400566(3.28%) | 417261(3.36%) | 413918(3.34%) |
| Total Unmapped Reads | 2015213(16.21%) | 1996354(17.03%) | 2119598(17.19%) | 2058561(16.87%) | 2239790(18.01%) | 2278692(18.41%) |

The list of total reads mapped to *G. raimondii* Genome

| Sample ID | Null | WT | IAU20 | IAU22 | IAO24 | IAO167 |
|---|---|---|---|---|---|---|
| Total Reads | 12434248(100%) | 11719314(100%) | 12329128(100%) | 12200881(100%) | 12435938(100%) | 12377822(100%) |
| Total BasePairs | 609278152(100%) | 574246386(100%) | 604127272(100%) | 597843169(100%) | 609360962(100%) | 606513278(100%) |
| Total Mapped Reads | 10033456(80.69%) | 9443822(80.58%) | 9999095(81.1%) | 9850187(80.73%) | 10088288(81.12%) | 9990549(80.71%) |
| perfect match | 5557554(44.7%) | 5193656(44.32%) | 5578388(45.25%) | 5462316(44.77%) | 5660773(45.52%) | 5620196(45.41%) |
| <=3bp mismatch | 4475902(36%) | 4250166(36.27%) | 4420707(35.86%) | 4387871(35.96%) | 4427515(35.6%) | 4370353(35.31%) |
| unique match | 9254531(74.43%) | 8700741(74.24%) | 9192172(74.56%) | 9081140(74.43%) | 9075324(72.98%) | 9043704(73.06%) |
| multi-position match | 778925(6.26%) | 743081(6.34%) | 806923(6.54%) | 769047(6.3%) | 1012964(8.15%) | 946845(7.65%) |
| Total Unmapped Reads | 2400792(19.31%) | 2275492(19.42%) | 2330033(18.9%) | 2350694(19.27%) | 2347650(18.88%) | 2387273(19.29%) |

**Supplementary Table S3.** RPKM vaules of APXs in different fibre stages of *G. hirsutum* TM-1.

| | GeneID | Root | Stem | Leaf | Petal | Anther | Stigma | Ovule 0 DPA | Fiber 10 DPA | Fiber 20 DPA | Ovule 10 DPA | Ovule 20 DPA |
|---|---|---|---|---|---|---|---|---|---|---|---|---|
| cAPX | Gh_A05G0863 | 224.13 | 404.83 | 261.51 | 94.50 | 86.86 | 279.83 | 119.64 | 833.48 | 66.35 | 214.00 | 165.58 |
| | Gh_D05G3875 | 257.64 | 281.18 | 237.57 | 70.68 | 52.46 | 234.30 | 84.25 | 432.22 | 72.16 | 170.20 | 167.05 |
| | Gh A08G1744 | 40.03 | 57.03 | 47.38 | 7.32 | 5.38 | 63.03 | 55.21 | 40.38 | 13.35 | 51.99 | 56.49 |
| | Gh_D08G2093 | 78.96 | 195.81 | 96.24 | 19.08 | 14.32 | 74.00 | 37.47 | 9.53 | 16.01 | 45.62 | 33.84 |
| | Gh_A08G1745 | 2.32 | 1.51 | 2.60 | 0.00 | 0.20 | 1.62 | 4.39 | 0.42 | 1.42 | 2.99 | 1.81 |
| | Gh_D08G2094 | 4.48 | 1.80 | 2.71 | 0.48 | 1.58 | 5.09 | 9.12 | 3.52 | 2.15 | 6.39 | 5.05 |
| | Gh_A08G1746 | 81.40 | 88.66 | 79.05 | 22.48 | 69.58 | 93.89 | 83.60 | 111.66 | 32.54 | 126.08 | 56.72 |
| | Gh_D08G2095 | 34.37 | 40.89 | 34.92 | 10.60 | 8.87 | 34.25 | 25.36 | 27.39 | 14.32 | 42.34 | 27.21 |
| | Gh_A04G0652 | 0.93 | 0.00 | 0.00 | 0.00 | 0.00 | 0.00 | 0.00 | 0.00 | 0.00 | 0.00 | 0.00 |
| | Gh_D04G1116 | 0.12 | 0.00 | 0.17 | 0.00 | 0.40 | 0.00 | 0.00 | 0.00 | 0.26 | 0.00 | 0.09 |
| | Gh_A13G2003 | 0.00 | 1.10 | 0.65 | 0.15 | 13.82 | 0.00 | 0.06 | 0.31 | 6.94 | 0.53 | 0.51 |
| | Gh D13G2402 | 0.00 | 0.16 | 0.33 | 0.00 | 11.59 | 0.00 | 0.13 | 0.00 | 0.00 | 0.54 | 0.00 |
| perAPX | Gh_A02G1648 | 10.61 | 3.93 | 4.95 | 12.37 | 0.91 | 3.42 | 1.16 | 0.51 | 2.95 | 2.11 | 1.10 |
| | Gh_D03G0074 | 4.63 | 2.02 | 2.73 | 6.83 | 0.61 | 2.07 | 0.60 | 0.44 | 1.08 | 1.40 | 0.20 |
| | Gh_A01G1388 | 0.38 | 0.40 | 0.55 | 0.26 | 1.61 | 0.13 | 0.16 | 0.40 | 0.00 | 0.15 | 0.22 |
| | Gh_D01G1632 | 1.34 | 0.13 | 0.55 | 0.91 | 2.42 | 1.04 | 2.56 | 0.07 | 0.00 | 1.79 | 0.00 |
| | Gh_A03G1812 | 35.18 | 41.98 | 36.48 | 9.23 | 17.72 | 16.45 | 26.10 | 17.45 | 29.84 | 26.53 | 24.69 |
| | Gh_D02G2245 | 25.52 | 17.44 | 29.85 | 7.54 | 13.85 | 11.53 | 25.09 | 12.62 | 17.36 | 16.69 | 20.04 |
| tAPX | Gh_A06G0383 | 1.65 | 0.75 | 3.77 | 1.99 | 0.45 | 2.53 | 1.20 | 1.09 | 0.37 | 1.04 | 0.26 |
| | Gh_D06G0413 | 0.60 | 0.88 | 1.88 | 2.59 | 0.10 | 2.29 | 1.46 | 0.84 | 0.50 | 1.41 | 0.43 |
| sAPX | Gh_A05G3726 | 29.19 | 35.22 | 44.31 | 7.85 | 10.13 | 45.48 | 27.00 | 41.48 | 39.33 | 35.35 | 17.75 |
| | Gh_D05G2244 | 23.01 | 25.59 | 30.28 | 6.63 | 4.73 | 29.88 | 22.40 | 50.03 | 24.32 | 37.02 | 14.28 |
| APX-R | Gh_A06G2046 | 5.88 | 1.78 | 15.00 | 4.81 | 1.14 | 5.71 | 2.98 | 1.54 | 0.83 | 1.58 | 2.64 |
| | Gh_D06G1049 | 0.80 | 1.01 | 2.07 | 0.54 | 0.27 | 0.65 | 1.34 | 5.49 | 0.35 | 0.37 | 0.55 |
| APX-L | Gh_A06G0270 | 11.28 | 0.31 | 8.16 | 2.42 | 0.50 | 5.02 | 5.96 | 1.31 | 0.98 | 5.21 | 2.55 |
| | Gh_D06G0293 | 9.30 | 0.21 | 9.69 | 2.83 | 0.63 | 3.83 | 6.10 | 1.10 | 0.98 | 3.60 | 5.23 |

cAPX, cytosolic APX; perAPX, peroxisomal APX; tAPX, thylakoid APX; sAPX, chloroplast stromal APX; APX-R, APX-R type of APX; APX-L, APX-L type of APX. GeneID indicated the gene ID number in the database *G. hirsutum* TM-1 (PRJNA248163)

**Supplementary Table S4.** List of all transgenic lines in different generations.

| Construct [a] | T0 NO. | T0 leaf [b] | T0 10 DPA fibre [b] | Inserted copies | Seed (T0) [c] | T1/2/3 NO. [d] |
|---|---|---|---|---|---|---|
| | 110 | 0.107 | 0.015 | 2 | Y | |
| | 111-1 | 0.88 | 1.326 | 1 | Y | OA15 |
| | 111-2 | 0.69 | | 1 | Y | |
| | 111-3 | 0.553 | | | Y | |
| | 111-4 | | | | | |
| | 112 | 0.863 | 0.992 | 1 | Y | OA16 |
| | 113-1 | 1.067 | 1.143 | 2 | Y | |
| | 113-2 | 0.847 | | | Y | |
| | 114-1 | | | 2 | | |
| | 114-2 | 0.493 | | | | |
| | 115 | | | | | |
| | 116-1 | 1.026 | | | | |
| | 116-2 | | | 1 | | |
| | 116-3 | 0.863 | 0.93 | | Y | |
| | 117 | | | 3 | | |
| | 118 | 0.676 | | 2 | Y | |
| | 119 | 0.898 | | 2 | Y | |
| 35S::GhAPX1(OA) 19/24 | 120-2 | 0.867 | 0.728 | 3 | Y | |
| | 121-1 | 0.837 | | | Y | |
| | 121-2 | 0.163 | | 3 | | |
| | 121-3 | 0.648 | 0.398 | 3 | Y | |
| | 122 | 1.176 | 0.842 | 1 | Y | OA17 |
| | 123 | 0.074 | 0.01 | | Y | |
| | 124 | | | | | |
| | 125 | 0.738 | 0.916 | 1 | Y | OA18 |
| | 126-1 | | | | | |
| | 126-2 | | | | | |
| | 127-1 | 0.575 | 1.077 | 1 | Y | |
| | 128-1 | 1.653 | 0.899 | 1 | Y | |
| | 128-3 | 2.034 | 0.782 | | Y | |
| | 129-1 | 0.033 | | 1 | Y | |
| | 129-2 | 2.191 | 1.024 | | Y | |
| | 130 | | 0.995 | 1 | Y | |
| | 131-1 | 0.832 | 0.871 | 1 | Y | |
| | 131-2 | | | | Y | |
| | 132-1 | 1.836 | | 2 | | |
| | 132-2 | 1.437 | | | | |
| | 133 | | | | | |
| | 135 | | | 5 | Y | |
| | 136 | | | | | |
| GhAPX1UTRi(IAU) 10/22 | 137 | | | 6 | Y | |
| | 138 | | | | | |
| | 139-1 | | 0.001 | 3 or 4 | Y | |
| | 139-4 | | 0.002 | | Y | |
| | 139-5 | | 0.002 | | Y | |
| | 140 | | | | | |
| | 141 | | 0.611 | 2 | Y | IAU19 |

| | | | | | |
|---|---|---|---|---|---|
| | 142 | | | | |
| | 143 | | | | |
| | 144-1 | 0.006 | 2 | Y | |
| | 144-2 | 0.002 | 2 | Y | |
| | 144-3 | 0.003 | 1? | Y | IAU20 |
| | 145 | 0.986 | 2 | Y | |
| | 146 | 0.01 | 2 | Y | IAU21 |
| | 147-1 | | | | |
| | 148-1 | | | | |
| | 149-1 | 0.007 | 6 | Y | |
| | 150 | | | | |
| | 151 | | | | |
| | 152 | 0.003 | 1 or 2 | Y | IAU22 |
| | 153 | 0.004 | 2 | Y | IAU153 |
| | 154-1 | | | | |
| | 155 | | | | |
| | 156-1 | | | | |
| GhAPX1ORFi（IAO）5/13 | 160-1 | | | | |
| | 161-1 | 1.062 | 1 | Y | IAO23 |
| | 161-3 | 1.013 | 1 | Y | |
| | 162 | | | | |
| | 163 | | | | |
| | 164 | | | | |
| | 165-1 | | | | |
| | 166-1 | | | | |
| | 167 | | 3 | Y | IAO167 |
| | 168 | | 2 | Y | IAO24 |
| | 169-1 | | | | |
| | 170 | 0.012 | 1 | Y | IAO25 |
| | 171 | 0.755 | 1 | Y | IAO26 |
| | 172 | | | | |

“a” 35S::GhAPX1 (OA) indicates overexpression of the *GhAPX1* construct; GhAPX1UTRi (IAU) indicates specific interference of the *GhAPX1* construct; GhAPX1ORFi（IAO）indicates suppression of the cytosolic APX construct. The ratio indicates the proportion of transgenic lines with setting seeds compared with all transgenic lines for one construct.

“b” Relative expression level of $GhAPX1A_T/D_T$ in the transgenic lines compared with WT by RT-qPCR.

“c” “Y” indicates the line used to collect seeds.

“d” Lines selected for further analysis of the T1/2/3 generation.

**Supplementary Table S5.** Fibre quality analysis with a HVI (High Volume Instrument) harvested from T3 plants grown in fields (2013).

| 2013 (T3) | UHML (mm) | UI (%) | Mic | Str (g/tex) | Elg (%) | MR | SFI |
|---|---|---|---|---|---|---|---|
| OA15 | 27.18±0.29bc | 85.93±0.64a | 5.81±0.09b | 27.03±0.91a | 6.65±0.06a | 0.86±0.006ab | 7.03±0.26c |
| OA16 | 26.41±0.22de | 84.65±0.35abc | 5.96±0.06b | 25.40±0.28bc | 6.60±0.00ab | 0.86±0.007ab | 7.65±0.21bc |
| OA17 | 26.77±0.22cd | 84.45±0.76abc | 6.31±0.10a | 26.08±1.00ab | 6.65±0.06a | 0.86±0.005a | 7.80±0.50bc |
| OA18 | 25.94±0.47ef | 84.45±1.17abc | 5.00±0.13d | 23.93±1.60de | 6.35±0.06c | 0.83±0.006d | 7.88±0.84abc |
| NG | 27.91±0.54b | 85.30±0.77ab | 5.33±0.01c | 25.65±0.76abc | 6.60±0.00ab | 0.84±0.005c | 7.23±0.34c |
| WT | 27.59±0.48b | 85.00±1.01ab | 5.37±0.10c | 25.05±0.6bcd | 6.53±0.05ab | 0.84±0.000c | 7.43±0.43bc |
| IAU20 | 28.52±0.16a | 85.18±0.64ab | 5.36±0.23c | 26.40±0.74ab | 6.60±0.00ab | 0.85±0.005bc | 7.25±0.26c |
| IAU21 | 26.16±0.21def | 84.65±0.92abc | 5.03±0.04d | 22.00±0.28f | 6.35±0.07c | 0.82±0.000d | 7.75±0.64bc |
| IAU22 | 28.42±0.37a | 84.80±1.07abc | 5.54±0.03c | 25.68±0.55abc | 6.63±0.05ab | 0.85±0.005bc | 7.40±0.43bc |
| **IAO24** | **25.63±0.99f** | **83.27±0.93bc** | **4.94±0.40d** | **23.57±0.25e** | **6.27±0.23c** | **0.82±0.012d** | **8.80±0.89a** |
| **IAO167** | **27.17±0.08bc** | **83.65±2.05c** | **4.70±0.04e** | **24.20±0.28cde** | **6.50±0.14b** | **0.82±0.000d** | **8.35±1.34ab** |

Mature fibres were harvested from experimental fields at Huazhong Agricultural University, Wuhan, Hubei province on one experiment plot in 2013 (mean ± sd, n=3). Three samples were collected from each line for the fibre quality measurements. UHML, Upper Half Mean Length refers to the mean length of the top half of the fibres; UI, uniformity index; Mic, Micronaire; Str, Strength; Elg, Elongation; MR, maturity ratio; SFI, short fibre index. Values with different letters (a, b, c, d, e and f) indicate significant differences (Duncan's multiple comparisons. $P < 0.05$) in one fibre quality index.

**Supplementary Table S6.** List of DEGs (Differentially Expressed Genes) screened by group comparison of the transgenic lines.

| Gene ID | log2Ratio((IAU20-22)/(Null-WT)) | Probability | log2Ratio((IAO24-167)/(Null-WT)) | Probability | log2Ratio((IA024-167)/(IAU20-22)) | Probability | Transcript Annotation |
|---|---|---|---|---|---|---|---|
| HSP | | | | | | | |
| Gorai.011G254900 | -1.37 | 0.64 | 1.75 | 0.81 | 3.12 | 0.88 | Kunitz family trypsin and protease inhibitor protein |
| Gorai.011G254400 | -1.56 | 0.54 | 1.71 | 0.75 | 3.27 | 0.83 | Kunitz family trypsin and protease inhibitor protein |
| Gorai.006G036600 | -4.07 | 0.33 | 4.86 | 0.91 | 8.93 | 0.92 | HSP20-like chaperones superfamily protein |
| Gorai.001G110000 | 0.22 | 0.12 | 3.42 | 0.82 | 3.21 | 0.80 | HSP20-like chaperones superfamily protein |
| Gorai.009G131100 | 0.22 | 0.27 | 1.81 | 0.83 | 1.59 | 0.81 | HSP20-like chaperones superfamily protein |
| Gorai.001G114100 | 0.26 | 0.48 | 1.64 | 0.87 | 1.38 | 0.85 | HSP20-like chaperones superfamily protein |
| Gorai.005G043700 | -0.16 | 0.39 | 1.47 | 0.86 | 1.63 | 0.87 | HSP20-like chaperones superfamily protein |
| Gorai.013G139100 | -0.28 | 0.50 | 1.33 | 0.84 | 1.61 | 0.87 | HSP20-like chaperones superfamily protein |
| Gorai.004G006500 | 0.20 | 0.43 | 1.31 | 0.85 | 1.10 | 0.83 | HSP20-like chaperones superfamily protein |
| Gorai.009G095100 | 0.13 | 0.32 | 1.20 | 0.83 | 1.07 | 0.82 | HSP20-like chaperones superfamily protein |
| Gorai.001G114300 | 0.15 | 0.32 | 1.05 | 0.80 | 0.90 | 0.76 | HSP20-like chaperones superfamily protein |
| Gorai.005G070000 | -0.30 | 0.49 | 1.04 | 0.80 | 1.34 | 0.83 | HSP20-like chaperones superfamily protein |
| Gorai.011G256600 | -0.45 | 0.44 | 1.35 | 0.79 | 1.81 | 0.82 | heat shock cognate protein 70-1 |
| Gorai.003G073700 | -0.38 | 0.47 | 1.01 | 0.77 | 1.39 | 0.81 | F-box family protein |
| Gorai.009G064800 | 5.17 | 0.95 | 0.89 | 0.28 | -4.29 | 0.93 | Eukaryotic aspartyl protease family protein |
| Gorai.008G203800 | -0.24 | 0.38 | 1.58 | 0.85 | 1.81 | 0.86 | Cysteine proteinases superfamily protein |
| Gorai.009G453600 | 0.04 | 0.10 | 1.72 | 0.81 | 1.68 | 0.80 | Chaperone DnaJ-domain superfamily protein |
| Gorai.009G042000 | -0.09 | 0.23 | 1.54 | 0.86 | 1.64 | 0.87 | 17.6 kDa class II heat shock protein |
| Gorai.010G097900 | -0.23 | 0.46 | 1.33 | 0.85 | 1.56 | 0.85 | 17.6 kDa class II heat shock protein |
| ion homeostasis | | | | | | | |
| Gorai.002G026400 | 0.20 | 0.34 | 1.36 | 0.82 | 1.16 | 0.80 | proton gradient regulation 5 |
| Gorai.013G258500 | -0.63 | 0.66 | 1.00 | 0.78 | 1.62 | 0.85 | histidine-containing phosphotransmitter 3 |
| Gorai.012G001300 | -0.11 | 0.16 | 2.56 | 0.87 | 2.66 | 0.88 | Heavy metal transport/detoxification superfamily protein |

| | | | | | | | |
|---|---|---|---|---|---|---|---|
| Gorai.010G249000 | 0.12 | 0.25 | 1.19 | 0.81 | 1.07 | 0.79 | Heavy metal transport/detoxification superfamily protein |
| Gorai.002G219600 | -0.79 | 0.68 | 1.17 | 0.81 | 1.96 | 0.86 | Heavy metal transport/detoxification superfamily protein |
| Gorai.007G372700 | -0.54 | 0.55 | 1.55 | 0.83 | 2.10 | 0.86 | gamma tonoplast intrinsic protein |
| Gorai.008G082300 | 2.02 | 0.69 | 2.83 | 0.81 | 0.82 | 0.58 | electron carriers |
| lipid metablism | | | | | | | |
| Gorai.003G093900 | 0.83 | 0.64 | 1.99 | 0.85 | 1.15 | 0.78 | Plant stearoyl-acyl-carrier-protein desaturase family protein |
| Gorai.005G038900 | -0.28 | 0.54 | 0.86 | 0.78 | 1.15 | 0.83 | lipid transfer protein 1 |
| Gorai.002G183100 | 0.64 | 0.61 | 1.81 | 0.85 | 1.17 | 0.79 | glycosylphosphatidylinositol-anchored lipid protein transfer 1 |
| Gorai.010G240200 | 1.05 | 0.80 | 1.36 | 0.84 | 0.31 | 0.53 | GDSL-like Lipase/Acylhydrolase superfamily protein |
| Gorai.010G002300 | -0.73 | 0.54 | 4.03 | 0.96 | 4.76 | 0.97 | GDSL-like Lipase/Acylhydrolase superfamily protein |
| Gorai.002G090200 | -0.33 | 0.32 | 1.68 | 0.81 | 2.01 | 0.83 | GDSL-like Lipase/Acylhydrolase superfamily protein |
| Gorai.010G202800 | 1.52 | 0.79 | 2.07 | 0.85 | 0.55 | 0.60 | 3-ketoacyl-CoA synthase 19 |
| Redox homeostasis | | | | | | | |
| Gorai.002G064200 | 1.48 | 0.77 | 2.20 | 0.85 | 0.72 | 0.67 | Ycf1 protein |
| Gorai.004G219800 | 0.43 | 0.52 | 1.55 | 0.83 | 1.12 | 0.79 | Ycf1 protein |
| Gorai.011G078900 | 0.41 | 0.48 | 1.40 | 0.81 | 1.00 | 0.75 | Ycf1 protein |
| Gorai.010G119900 | 0.65 | 0.67 | 1.36 | 0.83 | 0.70 | 0.70 | Ycf1 protein |
| Gorai.008G073700 | 0.72 | 0.70 | 1.14 | 0.80 | 0.42 | 0.58 | Ycf1 protein |
| Gorai.012G032000 | 0.42 | 0.62 | 1.44 | 0.85 | 1.02 | 0.82 | 0 |
| Gorai.012G162900 | 0.49 | 0.48 | 1.61 | 0.81 | 1.12 | 0.76 | squalene epoxidase 2 |
| Gorai.011G020200 | -0.01 | 0.08 | 2.00 | 0.86 | 2.01 | 0.86 | Quinone reductase family protein |
| Gorai.004G150600 | 0.36 | 0.49 | 1.25 | 0.81 | 0.89 | 0.74 | photosynthetic electron transfer D |
| Gorai.012G016200 | 1.45 | 0.67 | 3.76 | 0.93 | 2.32 | 0.86 | Peroxidase superfamily protein |
| Gorai.010G001900 | -1.27 | 0.35 | 2.62 | 0.78 | 3.89 | 0.83 | Peroxidase superfamily protein |
| Gorai.009G192200 | -1.00 | 0.53 | 1.49 | 0.76 | 2.49 | 0.82 | Peroxidase superfamily protein |
| Gorai.001G163400 | -0.21 | 0.43 | 0.84 | 0.77 | 1.04 | 0.82 | NADH dehydrogenase 5B |
| Gorai.007G322600 | -0.38 | 0.54 | 0.90 | 0.77 | 1.28 | 0.82 | metallothionein 3 |
| Gorai.007G175100 | 0.03 | 0.12 | 1.19 | 0.83 | 1.16 | 0.82 | glutathione S-transferase PHI 9 |
| Gorai.009G087600 | -0.22 | 0.32 | 1.29 | 0.80 | 1.51 | 0.81 | cytochrome-c oxidases;electron carriers |

| | | | | | | | |
|---|---|---|---|---|---|---|---|
| Gorai.008G030000 | -0.07 | 0.15 | -2.22 | 0.82 | -2.15 | 0.81 | cytochrome BC1 synthesis |
| Gorai.008G125300 | -0.83 | 0.61 | 1.03 | 0.74 | 1.86 | 0.82 | Cytochrome b561/ferric reductase transmembrane with |
| Gorai.009G233600 | 0.27 | 0.22 | 3.14 | 0.89 | 2.87 | 0.88 | Cupredoxin superfamily protein |
| Gorai.009G280000 | -0.32 | 0.25 | 1.87 | 0.79 | 2.20 | 0.80 | Cupredoxin superfamily protein |
| Gorai.004G227300 | -0.01 | 0.09 | -5.58 | 0.98 | -5.57 | 0.98 | ascorbate peroxidase 2 |
| Gorai.009G104500 | -7.97 | 1.00 | -8.15 | 1.00 | -0.17 | 0.19 | ascorbate peroxidase 1 |
| Gorai.004G227200 | 0.19 | 0.30 | -3.95 | 0.89 | -4.14 | 0.91 | ascorbate peroxidase 1 |
| Gorai.004G227100 | 0.05 | 0.16 | -4.87 | 0.97 | -4.92 | 0.97 | ascorbate peroxidase 1 |
| Gorai.008G027600 | 0.06 | 0.09 | 2.72 | 0.85 | 2.66 | 0.85 | 2Fe-2S ferredoxin-like superfamily protein |
| cell wall related | | | | | | | |
| Gorai.005G025000 | -0.09 | 0.23 | 0.94 | 0.79 | 1.03 | 0.82 | xyloglucan endotransglucosylase/hydrolase 16 |
| Gorai.013G016700 | -0.34 | 0.28 | 1.91 | 0.81 | 2.25 | 0.82 | TRICHOME BIREFRINGENCE-LIKE 33 |
| Gorai.010G194900 | -0.74 | 0.61 | 0.98 | 0.73 | 1.72 | 0.82 | TRICHOME BIREFRINGENCE-LIKE 33 |
| Gorai.010G171400 | 0.27 | 0.47 | 1.31 | 0.84 | 1.04 | 0.81 | Tetratricopeptide repeat (TPR)-like superfamily protein |
| Gorai.011G148900 | -0.20 | 0.38 | 0.84 | 0.76 | 1.04 | 0.80 | Tetratricopeptide repeat (TPR)-like superfamily protein |
| Gorai.005G194900 | -0.16 | 0.29 | 1.07 | 0.78 | 1.23 | 0.80 | Pollen Ole e 1 allergen and extensin family protein |
| Gorai.006G061200 | 0.31 | 0.35 | 1.72 | 0.82 | 1.41 | 0.79 | LOB domain-containing protein 42 |
| Gorai.008G056000 | 0.38 | 0.56 | 1.41 | 0.84 | 1.03 | 0.81 | LOB domain-containing protein 41 |
| Gorai.005G225000 | -0.21 | 0.22 | 1.64 | 0.79 | 1.85 | 0.80 | hydroxyproline-rich glycoprotein family protein |
| Gorai.011G137200 | 4.45 | 0.81 | -0.90 | 0.13 | -5.35 | 0.82 | homolog of carrot EP3-3 chitinase |
| Gorai.006G083000 | -0.27 | 0.24 | 1.88 | 0.80 | 2.15 | 0.81 | Glycine-rich protein family |
| Gorai.005G187500 | -1.13 | 0.80 | 0.27 | 0.47 | 1.39 | 0.83 | germin-like protein 7 |
| Gorai.004G061700 | -0.88 | 0.78 | 0.74 | 0.76 | 1.61 | 0.87 | FASCICLIN-like arabinoogalactan 7 |
| Gorai.001G131200 | 0.12 | 0.10 | 3.07 | 0.81 | 2.96 | 0.80 | FASCICLIN-like arabinogalactan-protein 11 |
| Gorai.007G374100 | -0.36 | 0.61 | 0.90 | 0.79 | 1.27 | 0.84 | FASCICLIN-like arabinogalactan-protein 11 |
| Gorai.006G078900 | -0.18 | 0.29 | 1.37 | 0.82 | 1.56 | 0.82 | chitinase-like protein 2 |
| Gorai.008G272000 | 5.82 | 0.87 | 0.03 | 0.06 | -5.79 | 0.87 | chitinase A |
| Gorai.008G272100 | 4.51 | 0.80 | 0.63 | 0.13 | -3.88 | 0.77 | chitinase A |
| Gorai.007G027100 | -0.66 | 0.22 | 3.39 | 0.86 | 4.05 | 0.88 | Bifunctional inhibitor/lipid-transfer protein/seed storage 2S |

| | | | | | | | |
|---|---|---|---|---|---|---|---|
| Gorai.008G237600 | 0.33 | 0.44 | 1.87 | 0.86 | 1.53 | 0.83 | Bifunctional inhibitor/lipid-transfer protein/seed storage 2S |
| Gorai.007G090100 | -0.41 | 0.53 | 1.55 | 0.85 | 1.96 | 0.87 | Plant invertase/pectin methylesterase inhibitor superfamily |
| stress response | | | | | | | |
| Gorai.011G023600 | 3.00 | 0.50 | 4.96 | 0.82 | 1.97 | 0.67 | Wound-responsive family protein |
| Gorai.005G206100 | 0.43 | 0.33 | 2.66 | 0.86 | 2.23 | 0.84 | Wound-responsive family protein |
| Gorai.009G174200 | 0.89 | 0.58 | 2.55 | 0.87 | 1.66 | 0.82 | Wound-responsive family protein |
| Gorai.005G206000 | 0.20 | 0.32 | 2.24 | 0.88 | 2.05 | 0.87 | Wound-responsive family protein |
| Gorai.005G205900 | 0.20 | 0.38 | 1.41 | 0.84 | 1.21 | 0.82 | Wound-responsive family protein |
| Gorai.003G134300 | 0.45 | 0.55 | 2.54 | 0.90 | 2.09 | 0.88 | Pathogenesis-related thaumatin superfamily protein |
| Gorai.008G044000 | 5.50 | 0.89 | -2.96 | 0.18 | -8.46 | 0.90 | pathogenesis-related gene 1 |
| Gorai.003G083600 | -0.21 | 0.19 | 2.07 | 0.81 | 2.28 | 0.81 | Disease resistance-responsive (dirigent-like protein) family |
| osmotic pressure | | | | | | | |
| Gorai.001G228900 | -1.09 | 0.54 | 2.32 | 0.85 | 3.40 | 0.89 | stomagen |
| Gorai.003G160400 | 5.49 | 0.87 | 1.44 | 0.20 | -4.05 | 0.83 | osmotin 34 |
| Gorai.007G338600 | -0.68 | 0.49 | 1.44 | 0.78 | 2.12 | 0.82 | osmotin 34 |
| Gorai.009G041800 | 0.77 | 0.48 | 2.31 | 0.82 | 1.54 | 0.76 | Mitochondrial substrate carrier family protein |
| Gorai.013G220800 | -0.28 | 0.42 | 0.96 | 0.76 | 1.25 | 0.80 | Mitochondrial substrate carrier family protein |
| Gorai.004G086000 | -0.14 | 0.28 | 2.05 | 0.88 | 2.19 | 0.89 | homolog of Medicago truncatula MTN3 |
| Gorai.011G202100 | 0.21 | 0.40 | 1.01 | 0.80 | 0.80 | 0.75 | cell wall / vacuolar inhibitor of fructosidase 1 |
| Gorai.011G202200 | -0.30 | 0.48 | 1.00 | 0.79 | 1.30 | 0.83 | cell wall / vacuolar inhibitor of fructosidase 1 |
| ribosomal protein | | | | | | | |
| Gorai.011G034100 | 0.48 | 0.65 | 1.25 | 0.84 | 0.77 | 0.75 | structural constituent of ribosome |
| Gorai.004G205100 | 0.44 | 0.58 | 1.10 | 0.81 | 0.66 | 0.70 | RNA polymerase subunit alpha |
| Gorai.013G136400 | 0.39 | 0.61 | 1.18 | 0.83 | 0.78 | 0.76 | ribosomal protein S8 |
| Gorai.005G167500 | 0.37 | 0.58 | 1.33 | 0.84 | 0.95 | 0.78 | ribosomal protein S19 |
| Gorai.009G350300 | 0.59 | 0.66 | 1.61 | 0.86 | 1.03 | 0.81 | Ribosomal protein L36 |
| Gorai.009G325200 | 0.26 | 0.51 | 1.20 | 0.84 | 0.94 | 0.78 | ribosomal protein L22 |
| Gorai.007G372600 | 0.13 | 0.33 | 1.13 | 0.83 | 1.01 | 0.82 | ribosomal protein L2 |
| Gorai.002G152200 | -0.34 | 0.32 | 1.83 | 0.82 | 2.16 | 0.84 | ribosomal protein L16 |

| | | | | | | | |
|---|---|---|---|---|---|---|---|
| Gorai.004G166700 | 0.20 | 0.42 | 1.11 | 0.83 | 0.91 | 0.78 | ribosomal protein L16 |
| Gorai.007G246200 | 0.19 | 0.24 | 1.75 | 0.82 | 1.56 | 0.79 | ribosomal protein L14 |
| Gorai.008G180700 | -0.46 | 0.28 | 2.50 | 0.84 | 2.96 | 0.85 | ribonuclease 3 |
| Gorai.001G025700 | 0.07 | 0.18 | 1.16 | 0.82 | 1.09 | 0.81 | nuclear poly(a) polymerase |
| Gorai.013G128500 | 0.16 | 0.21 | 1.73 | 0.81 | 1.57 | 0.79 | chloroplast ribosomal protein S15 |
| Gorai.007G083900 | -0.02 | 0.11 | 1.17 | 0.83 | 1.18 | 0.83 | |
| matebic pathway | | | | | | | |
| Gorai.013G053000 | -1.31 | 0.71 | 1.09 | 0.75 | 2.40 | 0.85 | unknown seed protein like 1 |
| Gorai.011G215100 | 0.29 | 0.53 | -0.83 | 0.76 | -1.12 | 0.82 | unknown seed protein like 1 |
| Gorai.011G033300 | 0.43 | 0.59 | 1.24 | 0.83 | 0.81 | 0.75 | SGNH hydrolase-type esterase superfamily protein |
| Gorai.007G129000 | 0.82 | 0.74 | 1.25 | 0.82 | 0.43 | 0.61 | ralf-like 33 |
| Gorai.009G410600 | -0.08 | 0.22 | -1.12 | 0.82 | -1.03 | 0.81 | poly(A) binding protein 7 |
| Gorai.004G103600 | 7.69 | 0.84 | #N/A | #N/A | -7.69 | 0.83 | plant natriuretic peptide A |
| Gorai.011G282700 | 1.14 | 0.80 | 0.63 | 0.67 | -0.50 | 0.62 | HXXXD-type acyl-transferase family |
| Gorai.004G085500 | -0.28 | 0.22 | 2.00 | 0.79 | 2.28 | 0.80 | Flavin-containing monooxygenase family protein |
| Gorai.009G019500 | 0.21 | 0.20 | 2.05 | 0.81 | 1.84 | 0.79 | early nodulin-related |
| Gorai.002G069500 | -5.83 | 0.83 | -5.82 | 0.83 | 0.01 | 0.08 | Dynamin related protein 5A |
| Gorai.013G006700 | -5.14 | 0.85 | -5.24 | 0.85 | -0.09 | 0.08 | Dynamin related protein 4C |
| Gorai.004G213300 | -0.51 | 0.57 | 0.94 | 0.75 | 1.45 | 0.82 | cytochrome P450, family 86, subfamily A, polypeptide 8 |
| Gorai.009G071700 | -2.62 | 0.55 | 3.28 | 0.88 | 5.89 | 0.94 | cytochrome P450, family 706, subfamily A, polypeptide 6 |
| Gorai.006G224400 | 0.54 | 0.23 | 3.00 | 0.81 | 2.46 | 0.77 | Cytochrome P450 superfamily protein |
| Gorai.001G177300 | 0.63 | 0.57 | 1.68 | 0.83 | 1.05 | 0.76 | ATP synthase subunit beta |
| Gorai.005G109000 | 0.25 | 0.40 | 1.35 | 0.83 | 1.10 | 0.80 | ATP synthase subunit beta |
| Gorai.005G217500 | 0.48 | 0.62 | 1.32 | 0.83 | 0.84 | 0.75 | ATP synthase epsilon chain |
| Gorai.007G054600 | -0.33 | 0.37 | 1.35 | 0.79 | 1.69 | 0.82 | alpha/beta-Hydrolases superfamily protein |
| Gorai.007G304400 | 0.62 | 0.62 | 1.57 | 0.83 | 0.96 | 0.74 | Adenine nucleotide alpha hydrolases-like superfamily protein |
| signaling transduction | | | | | | | |
| Gorai.004G123800 | -0.41 | 0.38 | 1.40 | 0.78 | 1.81 | 0.81 | SAUR-like auxin-responsive protein family |
| Gorai.005G071300 | -0.53 | 0.62 | 0.95 | 0.77 | 1.48 | 0.83 | S-adenosylmethionine synthetase family protein |

| | | | | | | | |
|---|---|---|---|---|---|---|---|
| Gorai.006G066200 | 0.49 | 0.50 | 1.49 | 0.81 | 1.01 | 0.75 | Protein kinase superfamily protein |
| Gorai.007G220200 | -0.73 | 0.62 | 1.13 | 0.78 | 1.85 | 0.84 | Gibberellin-regulated family protein |
| Gorai.009G182300 | -1.72 | 0.78 | 1.02 | 0.75 | 2.73 | 0.87 | ethylene-forming enzyme |
| Gorai.005G016900 | -0.56 | 0.61 | 0.88 | 0.74 | 1.44 | 0.82 | EF hand calcium-binding protein family |
| Gorai.011G257500 | 3.19 | 0.86 | 1.28 | 0.55 | -1.91 | 0.79 | Cysteine/Histidine-rich C1 domain family protein |
| Gorai.008G127100 | -0.57 | 0.68 | 0.64 | 0.72 | 1.21 | 0.83 | Calcium-binding EF-hand family protein |
| transcript factor | | | | | | | |
| Gorai.006G159000 | 0.47 | 0.58 | 1.75 | 0.86 | 1.28 | 0.82 | sequence-specific DNA binding transcription factors |
| Gorai.009G016700 | -0.16 | 0.34 | -2.88 | 0.90 | -2.72 | 0.89 | plant-specific transcription factor YABBY family protein |
| Gorai.002G148300 | -0.43 | 0.25 | 2.38 | 0.82 | 2.80 | 0.83 | PAR1 protein |
| Gorai.001G025500 | -0.02 | 0.12 | 0.99 | 0.81 | 1.01 | 0.82 | Mitochondrial transcription termination factor family protein |
| Gorai.009G323600 | -2.84 | 0.36 | 3.11 | 0.78 | 5.95 | 0.85 | Homeobox-leucine zipper protein family |
| Gorai.002G126500 | -0.33 | 0.51 | 1.22 | 0.83 | 1.56 | 0.84 | basic leucine-zipper 44 |
| unknown | | | | | | | |
| Gorai.003G017000 | 4.87 | 0.95 | 4.25 | 0.91 | -0.61 | 0.64 | Protein of unknown function (DUF677) |
| Gorai.003G120500 | -0.10 | 0.19 | 1.50 | 0.82 | 1.60 | 0.83 | Protein of unknown function (DUF579) |
| Gorai.007G055100 | -0.54 | 0.20 | 2.87 | 0.80 | 3.41 | 0.82 | Protein of unknown function (DUF579) |
| Gorai.002G163200 | -1.12 | 0.54 | 1.53 | 0.75 | 2.64 | 0.82 | Protein of unknown function (DUF579) |
| Gorai.013G137000 | -0.64 | 0.73 | 0.74 | 0.76 | 1.38 | 0.85 | Protein of unknown function (DUF579) |
| Gorai.007G256700 | 0.61 | 0.41 | 2.22 | 0.81 | 1.60 | 0.77 | Protein of unknown function (DUF1637) |
| Gorai.001G247300 | -0.58 | 0.42 | 1.51 | 0.77 | 2.09 | 0.81 | Plant protein of unknown function (DUF828) |
| Gorai.002G075100 | 7.80 | 0.85 | 4.70 | 0.41 | -3.10 | 0.75 | |
| Gorai.007G320400 | -4.26 | 0.89 | 0.15 | 0.25 | 4.42 | 0.90 | |
| Gorai.008G264100 | 6.90 | 0.75 | 7.39 | 0.81 | 0.49 | 0.34 | |
| Gorai.009G330800 | 1.56 | 0.37 | 4.97 | 0.91 | 3.40 | 0.86 | |
| Gorai.003G045900 | 1.79 | 0.42 | 4.83 | 0.90 | 3.04 | 0.83 | |
| Gorai.002G267200 | -0.04 | 0.07 | 3.58 | 0.81 | 3.63 | 0.80 | |
| Gorai.009G438000 | -2.56 | 0.57 | 2.83 | 0.85 | 5.39 | 0.93 | |
| Gorai.003G046000 | 0.34 | 0.35 | 2.83 | 0.90 | 2.49 | 0.88 | |

| | | | | | | |
|---|---|---|---|---|---|---|
| Gorai.009G041900 | -2.54 | 0.54 | 2.55 | 0.81 | 5.09 | 0.90 |
| Gorai.009G272400 | 0.19 | 0.16 | 2.51 | 0.82 | 2.33 | 0.81 |
| Gorai.013G053100 | -1.23 | 0.64 | 2.28 | 0.86 | 3.51 | 0.91 |
| Gorai.002G214400 | 1.37 | 0.68 | 2.27 | 0.82 | 0.90 | 0.65 |
| Gorai.011G293400 | 0.79 | 0.70 | 2.04 | 0.87 | 1.25 | 0.82 |
| Gorai.002G152800 | -0.42 | 0.34 | 1.96 | 0.83 | 2.38 | 0.85 |
| Gorai.005G262100 | -0.02 | 0.09 | 1.86 | 0.84 | 1.88 | 0.84 |
| Gorai.007G052300 | 0.61 | 0.70 | 1.75 | 0.87 | 1.15 | 0.82 |
| Gorai.011G014600 | -0.46 | 0.54 | 1.69 | 0.86 | 2.15 | 0.88 |
| Gorai.007G252600 | 0.25 | 0.44 | 1.58 | 0.86 | 1.33 | 0.84 |
| Gorai.006G093200 | 0.22 | 0.33 | 1.57 | 0.83 | 1.35 | 0.81 |
| Gorai.007G322200 | 0.42 | 0.63 | 1.57 | 0.86 | 1.15 | 0.83 |
| Gorai.003G182700 | 0.20 | 0.37 | 1.33 | 0.83 | 1.13 | 0.81 |
| Gorai.002G090500 | -0.56 | 0.61 | 1.33 | 0.83 | 1.89 | 0.86 |
| Gorai.007G303200 | -0.15 | 0.29 | 1.26 | 0.82 | 1.42 | 0.83 |
| Gorai.007G322100 | 0.37 | 0.61 | 1.26 | 0.84 | 0.90 | 0.78 |
| Gorai.012G100900 | 0.08 | 0.21 | 1.24 | 0.83 | 1.16 | 0.82 |
| Gorai.001G035600 | 0.25 | 0.42 | 1.15 | 0.81 | 0.90 | 0.75 |
| Gorai.007G116800 | -0.33 | 0.54 | -1.11 | 0.81 | -0.78 | 0.72 |
| Gorai.002G211000 | -0.87 | 0.40 | 2.16 | 0.80 | 3.03 | 0.84 |
| Gorai.013G079000 | -0.45 | 0.41 | 1.38 | 0.78 | 1.83 | 0.82 |
| Gorai.007G326100 | -0.69 | 0.64 | 0.82 | 0.71 | 1.52 | 0.81 |
| Gorai.007G235900 | -0.83 | 0.75 | 0.61 | 0.71 | 1.44 | 0.84 |
| Gorai.007G320800 | -1.07 | 0.77 | 0.29 | 0.47 | 1.36 | 0.81 |
| Gorai.001G276700 | -0.38 | 0.62 | 0.74 | 0.76 | 1.12 | 0.83 |
| Gorai.002G117600 | -0.02 | 0.10 | 0.99 | 0.79 | 1.01 | 0.80 |

The log2Ratio ((IAU20-22)/(Null-WT)) indicates the log2 value of the gene expression levels in the IAU20 and IAU22 groups compared with the control group; the log2Ratio ((IAO24-167)/(Null-WT)) indicates the log2 value of the gene expression levels in the IAU24 and IAU167

groups compared with the control group; the log2Ratio ((IA024-167)/(IAU20-22)) indicates the log2 value of the gene expression levels in the IAU24 and IAU167 groups compared with the IAU20 and IAU22 groups.

The probability indicates the probability of one gene being differentially expressed between two groups.

**Supplementary Table S7**. RPKM vaules of APX family members in 10 DPA and 20 DPA fibres of demosticated and wild *G. hirsutum*.

| Abbr. | GeneID | TM1_10 | Maxxa_10 | CRB252_10 | Coker315_10 | CascotL7_10 | yucTX2090_10 | yucTX2094_10 | yucTX2095_10 | palmeriTX665_10 | TM1_20 | Maxxa_20 | CRB252_20 | Coker315_20 | CascotL7_20 | yucTX2090_20 | yucTX2094_20 | yucTX2095_20 | palmeriTX665_20 |
|---|---|---|---|---|---|---|---|---|---|---|---|---|---|---|---|---|---|---|---|
| cAPX | Gorai.004G227100.1 | 10.2 | 16.7 | 32.7 | 26.1 | 27.8 | 66.6 | 40.1 | 38.6 | 23.6 | 25.2 | 22.9 | 0.0 | 9.1 | 16.8 | 89.2 | 39.5 | 0.0 | 66.5 |
| | Gorai.004G227200.2 | 2.8 | 5.0 | 16.7 | 11.8 | 18.0 | 20.5 | 3.2 | 15.8 | 6.0 | 3.4 | 1.2 | 0.0 | 2.4 | 4.1 | 13.2 | 3.2 | 0.0 | 12.5 |
| | Gorai.004G227300.3 | 11.7 | 14.5 | 53.8 | 51.4 | 56.1 | 55.4 | 14.7 | 42.9 | 16.7 | 11.2 | 7.6 | 0.0 | 13.1 | 15.8 | 53.1 | 9.3 | 0.0 | 56.0 |
| | Gorai.009G104500.1 | 198.2 | 246.5 | 531.9 | 530.8 | 373.8 | 342.0 | 203.2 | 313.6 | 194.5 | 150.0 | 169.3 | 86.1 | 115.2 | 153.5 | 314.0 | 145.5 | 220.0 | 317.7 |
| | Gorai.009G420500.1 | 0.0 | 0.0 | 0.0 | 0.0 | 0.0 | 0.0 | 0.0 | 0.0 | 0.0 | 0.0 | 0.4 | 0.0 | 0.0 | 0.0 | 0.0 | 0.0 | 0.0 | 0.2 |
| | Gorai.013G266100.1 | 0.3 | 0.1 | 0.1 | 0.2 | 0.0 | 0.1 | 0.2 | 0.3 | 0.3 | 0.2 | 0.0 | 0.0 | 0.0 | 0.6 | 0.5 | 0.0 | 0.4 | 0.3 |
| perAPX | Gorai.002G196800.1 | 0.1 | 0.2 | 0.1 | 0.0 | 0.0 | 0.0 | 0.0 | 0.1 | 0.1 | 0.1 | 0.0 | 0.0 | 0.2 | 0.0 | 0.1 | 0.2 | 0.0 | 0.1 |
| | Gorai.005G254100.1 | 4.8 | 6.0 | 7.9 | 8.6 | 6.4 | 12.5 | 6.2 | 11.1 | 5.8 | 4.3 | 5.6 | 2.0 | 3.9 | 2.4 | 14.0 | 9.0 | 8.4 | 10.8 |
| | Gorai.003G008200.1 | 0.5 | 0.6 | 0.6 | 0.4 | 0.3 | 0.3 | 0.6 | 0.5 | 0.4 | 0.7 | 0.2 | 0.3 | 0.4 | 0.0 | 1.4 | 0.9 | 0.8 | 0.5 |
| chlAPX | Gorai.009G246900.1 | 64.6 | 70.2 | 72.8 | 73.8 | 70.1 | 27.6 | 36.3 | 35.6 | 48.9 | 23.1 | 36.7 | 12.0 | 13.1 | 16.8 | 23.5 | 14.0 | 19.2 | 34.0 |
| | Gorai.010G051400.1 | 0.5 | 0.9 | 0.9 | 0.6 | 0.6 | 0.9 | 0.7 | 0.8 | 0.4 | 0.5 | 0.5 | 0.7 | 0.5 | 0.4 | 0.7 | 0.7 | 0.7 | 0.3 |

| | | | | | | | | | | | | | | | | | | | |
|---|---|---|---|---|---|---|---|---|---|---|---|---|---|---|---|---|---|---|---|
| APX-R | Gorai.010G115200.1 | 0.4 | 2.5 | 1.2 | 0.5 | 0.6 | 1.1 | 1.0 | 1.0 | 0.5 | 0.3 | 0.7 | 0.0 | 0.0 | 0.2 | 1.8 | 0.5 | 0.5 | 0.8 |
| APX-L | Gorai.010G038200.1 | 0.3 | 2.9 | 0.8 | 0.7 | 0.5 | 2.4 | 1.1 | 2.1 | 0.7 | 0.7 | 0.7 | 0.6 | 0.0 | 0.7 | 2.9 | 1.4 | 1.4 | 1.3 |

cAPX, cytosolic APX; perAPX, peroxisomal APX; tAPX, thylakoid APX; sAPX, chloroplast stromal APX; APX-R, APX-R type of APX; APX-L, APX-L type of APX. Data of Expression levels for wild cottons (yucTX2090, yucTX2094, yucTX2095 and palmeriTX665) and domestication cottons (TM1, Maxxa, CRB252, Coker315 and CascotL7) were downloaded from NCBI (SRA061240). "_10"or "_20" indicates the gene expression levels in 10 DPA or 20 DPA fibers.

**Supplementary Table S8**. RPKM vaules of APXs in different fibre stages of *G. raimondii*.

| | GeneID | 0 DPA | 3 DPA | 10 DPA | 20 DPA |
|---|---|---|---|---|---|
| | Gorai.004G227200.2 | 35.5101 | 10.4998 | 75.2364 | 45.0922 |
| | Gorai.004G227100.1 | 31.2071 | 9.75488 | 25.5556 | 22.6662 |
| | Gorai.004G227300.3 | 62.8232 | 21.8755 | 70.7763 | 77.6137 |
| | Gorai.009G104500.1 | 86.8625 | 26.3403 | 230.473 | 230.854 |
| | Gorai.009G420500.1 | 0 | 0 | 0 | 0 |
| cAPX | Gorai.013G266100.1 | 0.34089 | 0 | 3.02774 | 1.40049 |
| | Gorai.003G008200.1 | 0.375829 | 0.571451 | 1.82586 | 1.58229 |
| | Gorai.002G196800.1 | 0.720512 | 0.304589 | 0.580874 | 0.705325 |
| perAPX | Gorai.005G254100.1 | 31.7938 | 8.36749 | 15.1295 | 27.444 |
| | Gorai.009G246900.1 | 20.4657 | 8.90649 | 33.7845 | 18.1002 |
| chlAPX | Gorai.010G051400.1 | 8.43791 | 1.42891 | 3.03986 | 2.3913 |
| APX-R | Gorai.010G115200.1 | 0.619905 | 0.156482 | 6.9611 | 1.01954 |
| APX-L | Gorai.010G038200.1 | 2.2117 | 2.20223 | 6.40041 | 1.61945 |

cAPX, cytosolic APX; perAPX, peroxisomal APX; tAPX, thylakoid APX; sAPX, chloroplast stromal APX; APX-R, APX-R type of APX; APX-L, APX-L type of APX.

**Supplementary Table S9.** Classification of APX genes in different species according to the amino acid sequences.

| Species | Abbreviation | Total | Ⅰ | Ⅱ | Ⅲ | Ⅳ | Ⅴ |
|---|---|---|---|---|---|---|---|
| *Manihot esculenta* | Me | 12 | 3 | 3 | 5 | 1 | - |
| *Ricinus communis* | Rc | 7 | 2 | 2 | 1 | 1 | 1 |
| *Linum usitatissimum* | Lu | 10 | 3 | 4 | 2 | 1 | - |
| *Populus trichocarpa* | Pt | 11 | 4 | 3 | 2 | 1 | 1 |
| *Medicago truncatula* | Mt | 7 | 2 | 2 | 1 | 1 | 1 |
| *Phaseolus vulgaris* | Pvu | 7 | 1 | 3 | 1 | 1 | 1 |
| *Glycine max* | Gm | 10 | 1 | 6 | 2 | 1 | - |
| *Cucumis sativus* | Csa | 7 | 2 | 2 | 1 | 1 | 1 |
| *Prunus persica* | Ppe | 7 | 2 | 2 | 1 | 1 | 1 |
| *Malus domestica* | Mdp | 11 | 4 | 4 | 2 | 1 | - |
| *Fragaria vesca* | Fv | 7 | 2 | 3 | 1 | 1 | - |
| *Arabidopsis thaliana* | At | 8 | 2 | 2 | 2 | 1 | 1 |
| *Arabidopsis lyrata* | Al | 10 | 3 | 5 | 2 | 1 | - |
| *Capsella rubella* | Cr | 8 | 2 | 4 | 1 | 1 | - |
| *Brassica rapa Chiifu-401 v1.2* | Br | 10 | 4 | 2 | 2 | 1 | 1 |
| *Thellungiella halophila* | Th | 9 | 2 | 4 | 2 | 1 | - |
| *Carica papaya* | Cp | 6 | 2 | 2 | 1 | - | 1 |
| *Gossypium raimondii* | Gr | 13 | 6 | 3 | 2 | 1 | 1 |
| *Gossypium arboreum* | Ga | 13 | 6 | 3 | 2 | 1 | 1 |
| *Gossypium hirsutum* | Gh | 26 | 12 | 6 | 4 | 2 | 2 |
| *Theobroma cacao* | Tc | 6 | 1 | 2 | 1 | 1 | 1 |
| *Citrus sinensis* | Csi | 6 | 2 | 2 | 1 | 1 | - |
| *Citrus clementina* | Cc | 8 | 2 | 2 | 1 | 1 | 1 |
| *Eucalyptus grandis* | Eg | 8 | 3 | 2 | 1 | 1 | 1 |
| *Vitis vinifera* | GSVIVT | 7 | 2 | 3 | 1 | 1 | - |
| *Solanum tuberosum* | DMT | 6 | 1 | 2 | 1 | 1 | 1 |
| *Solanum lycopersicum* | Sl | 9 | 3 | 2 | 2 | 1 | 1 |
| *Mimulus guttatus v1.1* | Mg | 5 | 2 | 1 | 1 | 1 | - |
| *Aquilegia coerulea* | Ac | 7 | 3 | 1 | 1 | 1 | 1 |
| *Sorghum bicolor v1.4* | Sb | 8 | 2 | 2 | 3 | 1 | - |
| *Zea mays* | Zm | 8 | 3 | 2 | 3 | - | - |
| *Setaria italica* | Si | 8 | 2 | 2 | 3 | 1 | - |
| *Panicum virgatum v0.0* | Pvi | 18 | 4 | 5 | 7 | 2 | - |
| *Oryza sativa* | Os | 10 | 2 | 2 | 4 | 1 | 1 |
| *Brachypodium distachyon* | Bd | 9 | 2 | 2 | 3 | 1 | 1 |
| *Selaginella moellendorffii* | Sm | 6 | 1 | 2 | 2 | 1 | - |
| *Physcomitrella patens v1.6* | Ppa | 5 | 2 | 1 | 1 | 1 | - |
| *Chlamydomonas reinhardtii* | Cr | 3 | - | - | 2 | 1 | - |
| *Volvox carteri* | Vc | 3 | - | - | 2 | 1 | - |
| *Coccomyxa subellipsoidea C-169* | Csu | 5 | - | 2 | 2 | 1 | - |
| *Micromonas pusilla CCMP1545* | Mp1545 | 3 | - | - | 1 | 1 | 1 |
| *Micromonas pusilla RCC299* | Mp299 | 4 | - | - | 3 | 1 | - |
| *Ostreococcus lucimarinus* | Ol | 3 | - | - | 2 | 1 | - |

Ⅰ, cytosolic APX; Ⅱ, peroxisome APX; Ⅲ, chloroplast APX; Ⅳ, APX-R; Ⅴ, APX-L. “-” indicates that it doesn’t find the type of APX in the species.
